# Supplementary material for: Using sociotechnical theory to understand medication safety work in primary care and prescribers’ use of clinical decision support: a qualitative study
Source: BMJ Open. 2023 Apr 27;13(4):e068798. doi: 10.1136/bmjopen-2022-068798 (PMC10151989; doi:10.1136/bmjopen-2022-068798)
Supplement: Supplementary data [file bmjopen-2022-068798supp001.pdf]

MANCHESTER 1824 The University of Manchester | The University of Nottingham UNITED KINGDOM · CHINA · MALAYSIA | THE UNIVERSITY of EDINBURGH

**INTERVIEW SCHEDULE**

**Interviewer introduction:**

I am [....] and I am a researcher at The University of Manchester. We are carrying out a project looking at how the clinical decision support (CDS) software tool Optimise Rx® is introduced and used over time in general practices. We are particularly interested in the role of this tool in the safe prescribing and monitoring of medications as it incorporates 'prescribing safety indicators' [describe them]. We would like to find out more about the experiences of primary care staff, managers and software developers who have worked with the Optimise Rx® system in some way.

This interview will last for up to one hour, but if this is not your first interview with us it may take more like 30 minutes. During our time together I'd like to discuss your experiences of Optimise Rx® in practice, with a focus on medication safety practices. If this is not your first interview, I would like to revisit some of the things you said last time for an update, whilst also exploring any new topics that you want to raise today. I should remind you that the interviews are confidential. I'd like to audio record the discussion if that is okay with you; this is simply to help me capture all of the information that comes out of it. If you prefer, I can make written notes instead. You can ask for the audio-recorder to be switched off at any point during the interview. The recordings will be destroyed as soon as they are transcribed, the transcripts will be kept in a secure location for five years after the study is complete and then they will be destroyed.

Before we begin, I'd like to provide some ground rules for the discussion:

- You are being digitally recorded, so speak clearly;
- We will anonymise the transcript so that nobody or nowhere can be identified by name. However, please try to avoid naming specific people or locations;
- Everything discussed here is confidential. However, if you were to reveal anything that would be considered unlawful or anything that would place you at somebody else at risk of harm, we may have to report this to a clinical supervisor or manager.

Unless you have any questions for me, then we can begin.



Jeffries M, *et al. BMJ Open* 2023; 13:e068798. doi: 10.1136/bmjopen-2022-068798

**<u>Part one: background</u>**

*Can you tell me a little about your professional background and current place(s) of work?*

- Name and gender
- Profession
- Years of experience
- Current job title and place of work

*The CDS system Optimise Rx®*

- (practice staff) how long has Optimise Rx® been installed, where is it installed
- Was anything like Optimise Rx® installed previously
- At what point did you become involved with the CDS system



Jeffries M, *et al. BMJ Open* 2023; 13:e068798. doi: 10.1136/bmjopen-2022-068798

<u>**Part two: main discussion**</u>

**Questions chosen will depend upon how many interviews have been conducted with the participant and what past response(s) were, as multiple interviews may take place over time at 0-6 months, 6-18 months, and 18 months + post implementation of Optimise Rx®. Some members of staff may only be interviewed once or twice, at early or late time points.**

1. *Can you describe how Optimise Rx® was introduced /continues to be used into your workplace?*
    a. Who drove things forward and how, did they involve others
    b. Roles – you and management
    c. Allocation of resources and training/support for the tool – who, what, when
    d. What went well, what could be done better

2. *What do you think Optimise Rx® is there for and how it should it be used? (changing views?)*
    a. Focus participant on prescribing and monitoring safety, and the indicators
    b. Do your colleagues share your views?
    c. Do some people know more about the tool than others? What is the impact of this
    d. Do you think that the tool has actual or potential value / benefits for your work?

3. *Please describe how Optimise Rx® is used / continues to be used by you and your colleagues as part of your work, in the context of prescribing and monitoring safety.*
    a. Where possible focus attention on prescribing safety indicators
    b. Initial views/expectations versus reality of use
    c. Have you contributed to the CDS system
    d. Frequency and nature of interaction with the tool over time
    e. Who uses the tool and what outputs are generated, are some users different

4. *Can you tell me about how Optimise Rx® 'fits in' with your existing work? (over time)*
    a. Where possible focus attention on prescribing safety indicators
    b. Has the tool changed the way you or the team works?
    c. Has the tool changed the nature of yours or others work – type, volume, order
    d. Have you or others adapted your work to suit the tool, or vice versa
    e. Has the tool become an integral part of daily work? How was this achieved
    f. Do you (still) think that using the tool should be part of your role/duties and why?
    g. Do you (still) think that this CDS system should be used routinely across primary care?



Jeffries M, *et al. BMJ Open* 2023; 13:e068798. doi: 10.1136/bmjopen-2022-068798

5. *How well does Optimise Rx® work in your practice(s)?*
   a. Is the information produced by the CDS in relation to prescribing and monitoring safety: understood, timely, relevant, useful?
   b. Have the potential benefits of the tool been realised?
   c. Are there any problems with how well the CDS system works and how are these dealt with, e.g. technical issues, interface issues
   d. Has the system disrupted working relationships?
   e. Is any information collected to find out how well the CDS system is working for you and the team? Who is responsible and how is this information used
   f. Do you have any ways in which the team get together to talk about the CDS tool and how well it is working for you? Who is responsible for driving/organising this and what have been the outputs of such activity
   g. What is your opinion of the value of the system on your work, and does this differ to the wider team?
   h. Do you have confidence in the system – what gives you confidence or takes it away
   i. Do you have confidence in others using the system, are some people more skilled than others in using it and how does this affect things
   j. Example of where the CDS has improved / struggled to improve care?
   k. Do you support the use / ongoing use of the system?

**<u>Interviewer conclusion:</u>**

Concluding questions

- *Is there anything that you would like to talk about?*
- *Is there anything that you would like to go back and talk about?*

Switch off tape recorder

**[INTERVIEWER TO ARRANGE SUBSEQUENT INTERVIEW(S) IF REQUIRED AND PRACTICAL]**

Many thanks for taking the time to help us with this study. Your contribution has been extremely valuable. If you wish we can send you a copy of your interview transcript, and you can also request to receive a summary of the findings of this research study, just ask us. In the meantime please feel free to contact either myself or the other researcher(s) involved if you have questions in future.



Jeffries M, *et al. BMJ Open* 2023; 13:e068798. doi: 10.1136/bmjopen-2022-068798
