# Supplementary material for: Using sociotechnical theory to understand medication safety work in primary care and prescribers’ use of clinical decision support: a qualitative study
Source: BMJ Open. 2023 Apr 27;13(4):e068798. doi: 10.1136/bmjopen-2022-068798 (PMC10151989; doi:10.1136/bmjopen-2022-068798)
Supplement: Author's manuscript [file bmjopen-2022-068798.draft_revisions.pdf]

BMJ Open is committed to open peer review. As part of this commitment we make the peer review history of every article we publish publicly available.

When an article is published we post the peer reviewers' comments and the authors' responses online. We also post the versions of the paper that were used during peer review. These are the versions that the peer review comments apply to.

The versions of the paper that follow are the versions that were submitted during the peer review process. They are not the versions of record or the final published versions. They should not be cited or distributed as the published version of this manuscript.

BMJ Open is an open access journal and the full, final, typeset and author-corrected version of record of the manuscript is available on our site with no access controls, subscription charges or pay-per-view fees (http://bmjopen.bmj.com).

If you have any questions on BMJ Open's open peer review process please email info.bmjopen@bmj.com

BMJ Open

Using Sociotechnical Theory to Understand Medication Safety Work in Primary Care and Prescribers use of Clinical Decision Support: A Qualitative Study.

Journal:	*BMJ Open*
Manuscript ID	bmjopen-2022-068798
Article Type:	Original research
Date Submitted by the Author:	03-Oct-2022
Complete List of Authors:	Jeffries, Mark; The University of Manchester, NIHR Greater Manchester Primary Care Patient Safety Translational Research Centre; The University of Manchester, Centre for Pharmacoepidemiology and Drug Safety, Division of Pharmacy and Optometry, School of Health Sciences<br>Salema, Nde-Eshimuni; University of Nottingham, Centre for Academic Primary Care; The University of Manchester, NIHR Greater Manchester Primary Care Patient Safety Translational Research Centre.<br>Laing, Libby; University of Nottingham, Centre for Academic Primary Care<br>Shamsuddin, Azwa; University of Hull, School of Health Sciences<br>Sheikh, Aziz; University of Edinburgh, Division of Community Health Sciences<br>Avery, Tony; University of Nottingham, Primary Care<br>Chuter, Antony; University of Nottingham<br>Waring, Justin ; University of Birmingham<br>Keers, Richard; University of Manchester, Centre for Pharmacoepidemiology and Drug Safety
Keywords:	PRIMARY CARE, QUALITATIVE RESEARCH, Quality in health care < HEALTH SERVICES ADMINISTRATION & MANAGEMENT

SCHOLARONE™
Manuscripts

I, the Submitting Author has the right to grant and does grant on behalf of all authors of the Work (as defined in the below author licence), an exclusive licence and/or a non-exclusive licence for contributions from authors who are: i) UK Crown employees; ii) where BMJ has agreed a CC-BY licence shall apply, and/or iii) in accordance with the terms applicable for US Federal Government officers or employees acting as part of their official duties; on a worldwide, perpetual, irrevocable, royalty-free basis to BMJ Publishing Group Ltd ("BMJ") its licensees and where the relevant Journal is co-owned by BMJ to the co-owners of the Journal, to publish the Work in this journal and any other BMJ products and to exploit all rights, as set out in our licence.

The Submitting Author accepts and understands that any supply made under these terms is made by BMJ to the Submitting Author unless you are acting as an employee on behalf of your employer or a postgraduate student of an affiliated institution which is paying any applicable article publishing charge ("APC") for Open Access articles. Where the Submitting Author wishes to make the Work available on an Open Access basis (and intends to pay the relevant APC), the terms of reuse of such Open Access shall be governed by a Creative Commons licence – details of these licences and which Creative Commons licence will apply to this Work are set out in our licence referred to above.

Other than as permitted in any relevant BMJ Author's Self Archiving Policies, I confirm this Work has not been accepted for publication elsewhere, is not being considered for publication elsewhere and does not duplicate material already published. I confirm all authors consent to publication of this Work and authorise the granting of this licence.

For peer review only

Using Sociotechnical Theory to Understand Medication Safety Work in Primary Care and Prescribers use of Clinical Decision Support: A Qualitative Study.

Mark Jeffries (Corresponding Author)

NIHR Greater Manchester Patient Safety Translational Research Centre, Division of Pharmacy and Optometry, School of Health Sciences, Room 1.134 | Stopford Building | The University of Manchester | Oxford Road | Manchester | M13 9PT | 0161 275 3680

mark.jefferies@manchester.ac.uk

Nde-Eshimuni Salema [1, 2]

Libby Laing [1,3],

Azwa Shamsuddin [3,4]

Aziz Sheikh,[4]

Anthony J Avery[1, 2]

Antony Chuter [5]

Justin Waring[7]

Richard N Keers [1,7,8]

[1] NIHR Greater Manchester Primary Care Patient Safety Translational Research Centre, University of Manchester, Manchester Academic Health Sciences Centre (MAHSC), Manchester, United Kingdom.

[2] Centre for Academic Primary Care, School of Medicine, University of Nottingham, Nottingham, United Kingdom.

[3] School of Health Sciences, Cottingham Road University of Hull, United Kingdom.

[4]Usher Institute, University of Edinburgh, United Kingdom.

[5] 68 Brighton Cottages, Copyhold Lane, Lindfield, Haywards Heath, West Sussex, United Kingdom.

For peer review only

[6]School of Social Policy, Health Services Management Centre, University of Birmingham, United Kingdom.

[7] Centre for Pharmacoepidemiology and Drug Safety, Division of Pharmacy and Optometry, School of Health Sciences, University of Manchester, Manchester, United Kingdom. 0161 275 3680

[8] Pharmacy Department, Greater Manchester Mental Health NHS Foundation Trust, Manchester, United Kingdom.

Word Count 4741

For peer review only

For peer review only - http://bmjopen.bmj.com/site/about/guidelines.xhtml

ABSTRACT

Objective and Setting

The concept of safety work draws attention to the intentional work of ensuring safety within complex care systems. Clinical Decision Support (CDS) have been designed to enhance medication safety in primary care by providing decision-making support to prescribers. Sociotechnical theory understands that healthcare settings are complex and dynamically connected systems of fluid networks, human agents, changing relationships and social processes. This study aimed to understand the relationship between safety work and the use of CDS.

Design

The study took place across nine different general practices in England. Stakeholders included general practitioners and general practice-based pharmacists and nurse prescribers. Semi-structured interviews were conducted to illicit how the system was used by the participants in the context of medication safety work. Data analysis was thematic and conducted alongside data collection.

Results

Twenty-three interviews were conducted with 14 GPs, three nurse prescribers and three practice pharmacists between February 2018 and June 2020. Safety work was contextually situated in a complex network of relationships. Three interconnected themes were interpreted from the data: i) the use of CDS within organisational and social practices and workflows; ii) safety work and the use of CDS within the interplay between prescribers, patients and populations; and iii) the functionality and affordances embedded in CDS systems.

Conclusion

For peer review only

The use of sociotechnical theory here extends current thinking in patient safety particularly in the ways that safety work was co-constituted with the use of CDS alerts. This has implications for implementation and use to ensure that the contexts into which such clinical decision support systems are implemented are taken into account. Understanding how alerts can adapt safety culture will help improve the efficacy of CDS systems, enhance prescribing safety and help to further understand how safety work is achieved in primary care.

Strengths and Limitations

- This study reinforces the sense that primary care and medication safety operate as complex systems with multiple and interconnected parts
- A particular strength of this study is the focus upon sociotechnical theory, which enabled a nuanced understanding of the ways in which the CDS was utilised.
- This study had a longitudinal design with a variety of stakeholders from different professions interviewed over time across multiple geographical locations.
- A full range of different prescribers in different roles (GPs, nurses and pharmacists) were interviewed but there could have been greater breadth across general practices.
- Many practice staff were drawn from two of the four targeted CCG areas due to recruitment difficulties related to GP time and workload.

For peer review only

For peer review only - http://bmjopen.bmj.com/site/about/guidelines.xhtml

BACKGROUND

Healthcare services are increasingly interpreted as complex systems comprising fluid networks of people and technologies that interact in dynamic, non-linear and inter-dependent ways. [1-4] New thinking in the field of patient safety, often termed Safety-II, that focuses on 'work-as-done', rather than 'work-as-imagined', understands that safety is achieved less because people follow strict rules but rather because people adapt and respond to unanticipated events.[3 5] This idea departs from the 'measure and manage' orthodoxy of patient safety policies by attending to the situated practices of care work in the context of wider and inherently complex socio-cultural systems. [6 7] the concept of 'safety work' captures the idea that clinicians routinely need to engage in purposeful activities to actively create and restore safety in the context of dynamic and changeable systems.[8] Safety work has been understood from the concept of the multidimensional and dynamic safety culture of primary healthcare organisations. [9] This offers a way of exploring the interplay between system factors and also the purposeful behaviours of clinicians that goes beyond the reductionist and linear view of established safety perspectives, and instead foregrounds the combined influence of context and work as shaping clinical safety.

The implementation of Information Technology (IT) in health care settings has been explored from socio-technical perspectives that consider not only the ways technology is used and user characteristics, but the social and organisational context into which technology is embedded. [10 11] IT does not operate in isolation from social and organisational contexts and as such plays a potential role in both adding to system complexity and hence unsafe practice, and also contributing to the safety work of people within these systems. [1 12] Sociotechnical theory considers that people and technology are reciprocally and recursively related and as a consequence the outcomes of the relationships between the social, human agency and technology are considered as interdependent and not simply as the interactions between

For peer review only

homogenous unique elements. [13] Such an approach focuses upon the social processes involved in the use of the technology since it sees technological use and adoption as a social practice that may involve negotiation and conflict.

Extending this view, sociomateriality is a combination of the material properties of the technology with social processes. Sociomateriality considers that properties of the technology may be fixed therefore allowing for differences in use to be assigned to such social processes and contexts. [14] Technology has been conceptualised as offering affordances in which the material properties of the technology, through interaction with human agents, allow for the possibilities for certain actions. [15 16 17] In this way, technology has the possibility of both shaping human action and being shaped by different social processes and practices. [15] New rules and conventions may evolve in a dynamic interaction between the technology, users and contexts that then changes social processes and practices. [14 18]

Medications safety technologies

The World Health Organization's (WHO) Third Global Patient Safety Challenge: Medication Without Harm places medication safety as a global priority [19] Prescribing errors in general practice are an important and expensive cause of preventable safety incidents, illness, hospitalisations and deaths. [20] Medication safety across primary care operates within complex contexts that often involves collaboration between different general practice staff, between people and technology and is as such a fluid, negotiated, social process. [21 22 23] Prescribing errors have been considered to occur because of a multitude of error producing conditions including prescriber characteristics, patient characteristics, the working environment, the specific tasks and issues with technology. [24]

Prescribing safety in general practice is increasingly promoted through the use of specialist Clinical Decision Support (CDS)tools used to provide clinicians with clinical knowledge and

For peer review only - http://bmjopen.bmj.com/site/about/guidelines.xhtml

patient related information to improve the quality of prescribing and reduce medication error. [25 26] Previous evaluation of CDS systems focused upon those designed to improve care outcomes often in hospital settings. [25 27 28] CDS has been seen as moderately successful, but it is widely acknowledged that prescribers often override alerts due to high volumes and limited perceived value ('alert fatigue'), and particularly if they interrupt clinical workflow by arriving at inappropriate times in the consultation.[28-31] 'Success' or 'failure' in implementation of IT has in the past been attributed to material properties of the technology and interoperability, user characteristics, and organisational settings and processes. [32] It has however been suggested that discrete individual barriers and facilitators are less important than the complex interactions between those factor. [18 32] Engagement between the health professionals working in clinical settings and the technology may also inform understanding of the potential impact on changes in working practices, roles and responsibilities. [33 34] There has been little in the way of qualitative research that has utilised sociotechnical theory to understand how medication safety alerts might operate as part of the safety work undertaken by those working within primary care. This study aimed to apply sociotechnical theory to understand the use of CDS in the accomplishment of safety work in primary care, to understand how CDS transforms safety work, and how new forms of safety work are involved in the implementation and use of CDS.

METHODS

Study design

This qualitative study used semi-structured interviews with multiple stakeholders working as prescribers in general practices in England. The study took place across nine different general practices within four separate Clinical Commissioning Groups (CCGs), in the North West and East Midlands regions of England. The study was part of a wider qualitative longitudinal

For peer review only

process evaluation of interventions designed to improve prescribing safety in primary care. [35][36]

Sampling and recruitment of participants

The sampling was purposive and included people involved in prescribing medicines who had knowledge and experience of using CDS in their daily work in general practices within the four CCG areas, including GPs, general practice nurses and general practice-based pharmacists. Sampling and recruitment of general practices was facilitated through discussions with CCG managers. MJ approached potential practices between December 2017 and November 2019, by email or telephone and invited them to take part. In addition MJ visited CCG meetings of groups of practices. After each practice had indicated that their staff might be interested in taking part, the practice was visited by MJ to further explain the study and provide written information. Practices consented to take part in the study before individual staff were approached. Individual general practice staff were approached directly by telephone or email, or through liaising with the general practice manager. A total of 41 practices were approached; nine practices consented to take part and 32 either declined to take part or did not respond.. Practices commonly gave time and staffing commitments as reasons for not taking part. Each potential participant was provided with written information about the study and given a minimum of 24 hours to decide if they wished to take part. MJ then contacted those staff wanting to take part by telephone or email to arrange a convenient time for the interview. The interview process was two stage with follow-up interviews conducted approximately 12 months after the first interview with a convenience sample of different participants (n=7) in order to understand any changes that may have occurred and if any new themes were emerging.

Data collection

For peer review only - http://bmjopen.bmj.com/site/about/guidelines.xhtml

The semi-structured interview schedule was developed by MJ and RK drawing upon previous studies in medication safety in primary care. [11 33 37] This was designed to illicit how CDS was used by the participants in the context of medication safety work (see Appendix S1). In order to ensure it remained appropriate the interview schedule was reviewed iteratively throughout data collection. Interviews continued alongside data analysis in an iterative approach until data saturation was reached. We understood data-saturation as being achieved when we judged further interpretation of codes and themes would not provide further insights. [38] All interviews were conducted by MJ and took place face-to-face at the participants' usual place of work (general practice, n=19) or by telephone (n=4); all were digitally audio-recorded. Participants were offered a £20 shopping voucher per interview as acknowledgement of their time. Twenty-three interviews were conducted with 20 participants (14 GPs, 3 nurse prescribers and 3 practice pharmacists). The first interviews were conducted between February 2018 and November 2019 with follow-up interviews conducted between August 2019 and June 2020. Interviews ranged in duration from 13-41 minutes with a mean length of 31 minutes.

Data analysis

MJ led the data analysis, which was thematic and conducted alongside data collection following verbatim transcription. The analysis followed two parts: firstly, an inductive thematic approach informed by Braun and Clarke [39] and secondly a deductive template approach which involved developing a coding template from the first stage. [40] For the initial inductive thematic approach, MJ used QSR NVIVO 12 to organise the data and inductively coded early interviews to identify features, patterns, groups of codes and potential themes to build a preliminary framework for application to the data and to inform further data collection. These codes were discussed with the wider research team (RK NS LL AC ASha). These discussions led to the development of a further coding template that was applied to the

For peer review only

full data set, further refined and reworked. From this coding, the final themes as presented in the results were interpreted.

Patient and Public involvement

AC is a patient and public representative who was involved in all aspects of the study and is a co-author of the paper.

FINDINGS

Interpretative data analysis found a complex network of relationships between the technology, different users and the contexts in which the technology was utilised. Safety work and the use of CDS was contextually situated in this network and reciprocally interrelated with it.

Three broad, interconnected themes were interpreted from the interview data.

- The use of CDS within organisational and social practices and workflows;
- The use of CDS within the interplay between prescribers, patients and populations;
- Sociomateriality – the functionality and affordances embedded in CDS.

The use of CDS within organisational and social practices and workflows

For many prescribers the use of CDS reflected their established commitment to promoting safety within their practice which was described as illustrative of their a *'safety climate'*.

> *I think we are a safety conscious practice […] we don't want mistakes to happen so therefore creating a safe climate where you feel you are doing things safety is actually really important to us as a practice* ***GP12***

Organisational culture varied. One GP reflected upon these variations across different sites of the group practice in which they worked. At one site had a strong focus upon being *'traditionally good prescribers'* who had '*work(ed) very hard […] on the prescribing* ***(GP3)***.

For peer review only - http://bmjopen.bmj.com/site/about/guidelines.xhtml

Such 'good' prescribing was seen to be about stability which **(GP3)** suggested '*creates much improved prescribing'* in contrast to another site of their group practice which, they said, had high staff turnover. This was reflected in the number of CDS alerts seen. Having a '*safety system'* was a way of avoiding many alerts. A safety culture that included medication reviews and which had been '*done …for so long that it's relatively slick',* was perceived as conducive to utilising alerts from CDS systems in a way that avoided being overwhelmed.

> *Medication reviews I do here. This probably might explain why we've got less (alerts) here. We do a six month or annual review on people that are only on two things here, and we've done it for so long that it's relatively slick. Whereas other places where they've not had review I'm going into a jungle almost of interactions and possible problems. They've never had any safety system before, so there's lots of alerts will go on.* ***GP3***

Organisational practices involved workflows where the use of the system was most pertinent in medication reviews. The following GP utilised CDS mostly when undertaking repeat prescribing. This was perceived to lead to disregarding alerts because '*the patient's not in front of you'* and because of the volume of prescriptions that needed repeat authorisation.

> *I don't often use it (CDS support) in consultations, because I suppose that's one of the problems of it, […] the time it crops up in workflows most, is when you're signing today's 60 repeat prescriptions, so the patient's not in front of you, but there's a medication review overdue, […] (the alert) flashes up and tells you that something minor could need changing, and you sort of let it go.* ***GP13***

The use of CDS alerts within the interplay between prescribers, patients and populations.

The characteristics of patients and roles of prescribers impacted upon the CDS use for medication safety. Users with differing roles in general practice might not use CDS in the

For peer review only

same way. The wider demographics of patient groups and broader holistic needs of individual patients were considered relevant to how prescribing would be prioritised and how CDS was used. For one GP, changes to specific individual medications as alerted by CDS was not the biggest issue for patients. .

…if you've got a patient with 20 different drugs, […] they've just lost their job, they're about to become homeless, they've got epilepsy, depression, anxiety, diabetes that's out of control, they've got 15 appointments, but they've missed the last ten, and they've got a safeguarding issue with their child [...] There are much, much bigger issues for them. ***GP5***

Individual patient needs, particularly for patients with complex medical problems or multi-morbidity, could impact upon the ways in which CDS alerts were responded to by prescribers. This GP suggested that it was difficult to respond to alerts if patients were '*are on long lists of medication already'* ***GP14***

Alerts were rejected if the prescriber disagreed, and were thought inappropriate to the patient because of other morbidity, frailty or their social situation. Safety work was thus accomplished by the prescribers balancing alerts against their own knowledge and understanding of individual patient circumstances. In this specific case, leaving a patient with epilepsy without medication as the alert had suggested was perceived as less safe.

Sometimes it's hazardous not to prescribe [...] some of the epilepsy drugs you can't give them safely because they're dangerous drugs; and it's telling you all these warnings, but you've got to give something because it would be more dangerous to leave somebody fitting […] somebody with bad COPD (chronic obstructive pulmonary disease) and they're on a host of other drugs, there really isn't an antibiotic that you can give that

For peer review only - http://bmjopen.bmj.com/site/about/guidelines.xhtml

doesn't have a risk of cardiac arrhythmia or something like that; (but) , I can't leave them without the antibiotic [...] we've got to give something. ***GP1***

Safety work and prescribing was undertaken differently by different prescribers. This nurse considered that whilst they had been prescribing for 11 years it was generally felt amongst nursing staff that prescribing was an "*extended role*". This led to a lack of confidence, a fear of making mistakes and the seeking of support in the use of CDS.

I think the way nurses think because we see it as an extended role, it's not part of this. I think there's that element of it and we're always worried, we're always covering our back all the time. A doctor said to me once that it's the mind set of how we're trained, nurses are trained to question, have I done something wrong, and the doctors don't think like that. So it's how we think, scared to do something wrong. ***Nurse 1***

In contrast, the use of CDS was seen as being dependent upon experience and expertise. This GP suggested that it was easy to use and decide whether to accept alerts or not because they had many years' experience and was the prescribing lead for the practice. They suggested that they were more likely to rely upon their own experience and knowledge than less experienced prescribers who might be more likely to rely upon alerts.

So, I think from my personal perspective having been a GP for a long time, I'm also the prescribing lead for the practice […] So, obviously, that relies on your own experience, skills, confidence, that sort of thing. I think probably as maybe one of the relatively older GPs, […] having a reasonable amount of experience and confidence in what you're doing, probably means that you cancel it off more often than a younger one. ***GP14***

For this GP there was a clear contrast between how they undertook safety work using CDS and how that was conducted by their practice pharmacist. Practice pharmacists reported that they saw patients for longer appointment times and would be more likely to be dealing with

For peer review only

medication reviews for patients with long-term conditions. Their contrasting work practices were related to differences in response to CDS alerts with the GP less likely to be methodical and more likely to not change medications in case the patient complained.

> *…it's been interesting seeing how our pharmacist works, now that (they are) seeing patients and doing (their) own medication reviews. (They are) much more thorough and […] will challenge people a lot more and say, look you've been on this thing, I'm not sure you necessarily need that or you may be need something slightly different, and will go through it in a more methodical fashion. I think we do probably in our consultations, sort of, go through things a little bit more quickly.* ***GP4***

Sociomateriality – the functionality and affordances embedded in the CDS.

CDS was used within Electronic Health Record (EHR) systems so the usability and functionality of it was dynamically linked to other databases and clinical systems that could provide alerts. As a consequence this impacted upon how CDS was used, how users interacted with it, and how the alerts were responded to. Part of this was associated with a frustration with functionality and this led to perceptions about what different technologies could or could not do, or what could or could not be done with different technologies. Prescribers talked of CDS when embedded with one particular clinical system *'failing to launch' …'it's just so many more clicks on particular things of your screen'* ***GP12*** or '*the number of warnings that comes up in (name of clinical system) is far too many'* ***GP13.*** This was said to mean *'it slows down and frustrates you as you're doing an important task but at the same time you're not getting the advice from it as well.'* ***GP13***. Functionality in this way was not just about non-use or abandonment but about how that made users perceived the system and felt about using it, which then in turn led to a negative response. Such frustration with the functionality could lead to important medication safety issues.

For peer review only - http://bmjopen.bmj.com/site/about/guidelines.xhtml

*We had a significant event where a medication was prescribed to a patient that shouldn't have been. We know that at least one of the factors in that was the fact that in the set up process of (the clinical system) different doctors chose different security settings for medicines safety…. **GP13***

The perception that CDS was not providing accurate and useful alerts through the lack of functionality could mean that the system was ignored and therefore safety work potentially compromised.

*Maybe we just have lots of very complicated patients and the poor system is just having a wobbly because it's cross referring this medication against all these other drugs and all these conditions it perceives the patient to have. The problem is if it's digging out old conditions and old medications **GP6***

In contrast, some participants described how CDS provided affordances for prescribers to undertake safety work. This GP considered the alerts not intrusive, easily overridden and providing useful and sensible safety advice. Such safety advice was seen as important since it could mitigate against safety problems for patients in the future.

*I don't find them too intrusive really. I mean it is another thing that happens that didn't happen before but it's as we discussed before, it was they are relatively easy to override and the suggestions that are being made generally feel quite sensible […] And if it's a safety thing, then generally I've been quite pleased that it's happened because it's given me an opportunity to make a change before potentially something that might cause me a problem later down the line **GP4 (Follow-up interview)***

CDS was seen as providing prompts to safer prescribing that challenged prescribers to think about what they were about to prescribe. This could take the form, as described by this GP of avoiding a prescribing error.

For peer review only

... today it's prompted me not to prescribe the wrong dose of oxycodone. So off a picking list I was close to picking the wrong strengths of oxycodone. And that came up with an (CDS) prompt which was great. So I've generally found it really helpful. [...] , so I was very happy to have something held up in front of my face going, are you sure about that?. ***GP4***

DISCUSSION

The use of CDS for medication safety in primary care was seen to be contextually situated in the social practices of safety work. This builds upon understandings of healthcare settings as complex systems with different characteristics including people, tasks and technology. [3] In this study, safety work was accomplished within safety cultures, which varied for patients and prescribers and therefore shaped their particular forms of engagement with the CDS and safety work. The use of sociotechnical theory here extends current thinking in patient safety particularly in the ways that safety work was co-constituted with the use of CDS alerts.

Safety work in primary care

The safety work undertaken by prescribers when using CDS was often shaped by the perceived health needs and broader characteristics of the patient. Where CDS support was less easy to accommodate to the holistic practices of prescribers, in terms of their relationship with patients, it was typically underutilised. Specifically, for patients with multiple and complex care needs, prescribers suggested it was necessary to consider factors other than prescribing, and relatedly the existing CDS system presented functional barriers to working with patients on multiple medicines. A recent systematic review and meta-analysis of medication harm across health settings found the highest incidence of medication harm in patients with high comorbidity and related polypharmacy. [41] It has previously been suggested that decision-making is made more complicated for clinicians when dealing with patients with multi-morbidity. [42 43] As such, the

For peer review only - http://bmjopen.bmj.com/site/about/guidelines.xhtml

under-utilisation of CDS might be seen as stemming from a lack of appreciation amongst designers and policy-makers about how prescribing safety is shaped by the particular relationship between prescriber and patient, in the context of the former's prevailing safety practices and the latter's particular health needs.

We found that the utilisation of CDS for safety work within primary care was linked to the functionality and usability of the technology and significantly how these related to the perceived needs of the patient and the associated approach to medical decision-making. That is, GPs' safety work often involved ignoring alerts where they were perceived as inaccurate, overwhelming, or irrelevant to the complexity of the decision-making for a given patient. Although our study did find that in other relational contexts, i.e. with less complex patients, or where there was more time available such as in consultations with practice pharmacists, CDS did provide prescribers with valued and useful alerts that were seen as enhancing their safety work.

CDS alerts afforded the possibility of making prescribing changes but were only acted upon if the prescriber considered it appropriate within the given contexts of their practice. The system provided prompts and challenges in the form of alerts, which could be accepted, and acted upon in different ways, or ignored and thus there was a recursive relationship between people and the technology. [13]Responding to CDS alerts was therefore, not a linear and binary process but one that was fluid and contextually bound. Prescribers' responses to safety alerts were based upon their expertise and knowledge. Previously Swinglehurst [23] found that safety work in repeat prescribing was embedded in different types of organisational structures. This study reinforces the sense that primary care and medication safety operate as complex systems with multiple and interconnected parts. [1] We found that the organisational culture and what prescribers referred to as a 'safety climate' or 'safety culture' of particular importance with organisational practices such as workflows and tasks shaping the use of CDS. This reinforces that safety work

For peer review only

is a social process operating within multidimensional and dynamic systems.[8 9 44] As well as organisational processes shaping the use of CDS, CDS alerts shaped the way prescribers accomplished safety work as they variously adapted their clinical practices and responded differently to them. [1 3] This further highlights how safety work requires a flexible approach that is adaptive and responsive to different contextual situations and does not over rely upon technology. [23]

Strengths and Limitations

A particular strength of this study is the focus upon sociotechnical theory, which enabled a nuanced understanding of the ways in which the CDS was utilised. By using this theoretical lens we could further understand how the CDS alerts and those already embedded in the EHR were utilised within the context of organisational culture, and prescribers workflows. This builds upon the sociotechnical theory particularly that around the concept of affordances within technology that enable human actions.[15 16] Additionally, a further strength was the longitudinal design with a variety of stakeholders from different professions interviewed over time across multiple geographical locations. A full range of different prescribers in different roles (GPs, nurses and pharmacists) were interviewed but there could have been greater breadth across general practices; many practice staff were drawn from two of the four targeted CCG areas due to recruitment difficulties related to GP time and workload. Furthermore interviewing patients to gain their perspective may well have been useful. This study also relied solely upon interview data and may have benefited from observations or other ethnographic approaches that might have uncovered 'work-as-done'. [5]

Implications for future research, policy and practice.

CDS systems for medication safety have been utilised in primary care with varying success. [27 30 45] Since CDS systems operate within other technology such as the EHR it may be important

For peer review only - http://bmjopen.bmj.com/site/about/guidelines.xhtml

to understand how different technologies interact and offer different ways people can engage in safety activities. Understanding open systems and the complexity in healthcare has been highlighted as important in health care evaluation. [1 46] Investment into CDS systems needs to be judged against how those systems will be used not just from a functionality aspect but also from the view of who the users will be and for which individual and groups of patients they will utilising the system for. How CDS are utilised for patients with multi-morbidity and polypharmacy will be an important consideration here. An important finding of our study was that functionality was linked to patient characteristics and prescriber motivations in that where multiple alerts were received owing to patients having polypharmacy the system was then underutilised and the alerts could be ignored. Since this group of patients is at most risk from a prescribing error, this has important implications for practice. Multidisciplinary reviews of patients with polypharmacy have been found useful in other contexts including care homes. [47 48]

There is a recursive interrelationship between CDS alerts and safety culture in that CDS can enhance safety culture but may also be utilised differently within existing safety cultures as prescribers adapt their practices. [1 3] Further research could valuably explore how technology to enhance medication safety can shape organisational safety work and not just be utilised within a safety climate but be instrumental in building that safety culture. Understanding how alerts can adapt that safety culture and how prescribers may adapt to the alerts to form new emerging safety cultures is a challenge that will help improve the efficacy of CDS systems, enhance prescribing safety and help to further understand how safety work is achieved in primary care.

CONCLUSIONS

Sociotechnical theory enabled us to understand how the use of CDS alerts for medication safety in primary care is context bound and dependent upon a complex and reciprocal network of relationships between the technology, different users and the places and spaces in which the technology was utilised. Where prescribers perceived their practices to have a

For peer review only

safety culture this could impact upon the utilisation of CDS. The use of CDS for medication safety work was dependent upon who responded to alerts and for which patients the alerts were received. An important finding here is how the alerts were utilised for patients with multimorbidity and polypharmacy. Prescribers adapted their practices in response to alerts. This has implications for the use of decision support to ensure that the contexts into which it is implemented and the ways in which safety work is accomplished are taken into account.

Contributions

MJ, RK, AA and JW advanced the idea for the study. MJ collected the data. MJ led on data analysis with contributions from NS, LL, AC, RK and ASHA. MJ drafted the manuscript which was reviewed and commented upon by all authors. All authors approved the final manuscript.

Funding

This study was part of the NIHR PRoTeCT Programme grant. PRoTeCT is funded by the NIHR Programme Grants for Applied Research Programme (1214-20012). The views expressed are those of the author(s) and not necessarily those of the NIHR or the Department of Health and Social Care.

Additionally MJ, RK, AA, LL and NS received funding from the National Institute for Health Research through the Greater Manchester Patient Safety Translational Research Centre (NIHR Greater Manchester PSTRC) grant number PSTRC-2016-003.

Declaration of conflicting interests

The authors declare there is no conflict of interest.

Ethical approval and consent to participate

For peer review only

For peer review only - http://bmjopen.bmj.com/site/about/guidelines.xhtml

Ethical approval for the study was granted by the University of Manchester Research Ethics Committee. (Ref: 2017-0842-3977). Further approval to conduct the research was granted by the NHS Health Research Authority (IRAS project ID: 233079) and by the research and development offices for the NHS participating regions. General practices gave written informed consent for their staff to be approached to take part in the study. All interview participants gave written informed consent to take part in the study, and for interviews to be audio-recorded, transcribed verbatim and for anonymised quotations to be used in publications.

Data availability statement

This is a qualitative study confined to relatively small groups of health care professionals in specific roles, particularly GP staff. Making the full transcripts publicly available could therefore potentially lead to the identification of participants. Our ethics approval was granted based on the anonymity of the individuals consenting to participate and specifically referred to only anonymised quotations being used in reports. As such the participants did not consent to full their transcript being made publicly available. Therefore, data cannot be made publicly available.

REFERENCES

1. Greenhalgh T Papoutsi C. Studying complexity in health services research: desperately seeking an overdue paradigm shift. *BMC Medicine* 2018;16:95.

2. Lipsitz LA. Understanding health care as a complex system. *JAMA* 2012;308(3):243-244.

3. McNab D, McKay J, Shorrock S, *et al.* Development and application of 'systems thinking' principles for quality improvement. *BMJ Open Quality* 2020;9e000714.

4. Pype P, Mertens F, Helewaut F, *et al.* Health care teams as complex adaptive systems: understanding team behaviour through team members perception of interpersonal interaction. *BMC Health Services Research* 2018;18:570.

5. Hollnagel E, Wears RL, Braithwaite J. From Safety-I to Safety-II: A White Paper. The Resilient Health Care Net. Published simultaneously by the University of Southern Denmark, University of Florida, USA, and Macquarie University, Australia 2015

6. Rowley E, Waring J. *A socio-cultural perspective on patient safety.* Boca Raton, FL, London, NewYork: CRC Press, 2017

7. Wears RL, Sutcliffe KM. *Still not Safe: Patient Safety and The Middle-Managing of American Medicine.* New York: OUP, 2020.

8. Daker-White G, Hays R, Blakeman T *et al*. Safety work and risk management as burdens of treatment in primary care : insights from a focused ethnographic study of patients with multimorbidity. *BMC Family Practice* 2018;19(155).

9. Kirk S, Parker D, Claridge T, *et al.* Patient safety culture in primary care: developing a theoretical framework for practical use *Quality and Safety in Health Care* 2007;16:313-320.

10. Greenhalgh T, Stones R, Swinglehurst D. Choose and Book: a sociological analysis of 'resistance' to an expert system. *Social Science & Medicine* 2014;104:210-19.

11. Jeffries M, Phipps D, Howard RL, *et al.* Understanding the implementation and adoption of an information technology intervention to support medicine optimisation in primary care: qualitative study using strong structuration theory. *BMJ Open* 2017;7:e014810

12. Timmermans S, Berg M. The Practice of Medical Technology. *Sociology of Health and Illness,* 2003;25, 97-114.

For peer review only

For peer review only - http://bmjopen.bmj.com/site/about/guidelines.xhtml

13. Orlikowski WJ, Scott SV. Sociomateriality: challenging the separation of technology, work and organization. *Academy of Management Annals* 2008;2(1):433-474.

14. Leonardi PM. 'Materiality, Sociomateriality and Sociotechnical systems: What do these terms mean? How are they different? Do we need them?' in Leonardi PM, Nardi BA, Kallinikos J. eds. *Materiality and Organizing: Social Interaction in a Technological World.* Oxford: OUP, 2012 pp.25-48.

15. Hutchby I. Technologies, texts and affordances. *Sociology* 2001;*35*(2):441-456.

16. Petrakaki D, Klecun E, Cornford T. Changes in healthcare professional work afforded by technology: The introduction of anational electronic patient record in an English hospital. *Organization* 2016;23(2):206-226.

17. Petrakaki, D, Waring J. Barber N. Technological affordances of risk and blame: the case of the electronic prescription service in England. *Sociology of Health and Illness* 2014;36(5):703-718.

18. Klecun E. Transforming healthcare: Policy discourses of IT and patient-centred care. *European Journal of Information Systems* 2016;25:64-76.

19. World Health Organization Medication Without Harm - Global Patient Safety Challenge on Medication Safety. Geneva: WHO, 2017

20. Elliott RA, Camacho E, Jankovic D, *et al.* Economic analysis of the prevalence and clinical and economic burden of medication error in England. *BMJ Quality & Safety* 2021;30:96–105.

21. Allen D, Braithwaite J, Sandall J *et al.* Towards a sociology of healthcare safety and quality. *Sociol. Health Illn*. 2016; 38(2):181-197.

For peer review only

22. Jeffries M, Gude WT, Keers RN, *et al.* Understanding the utilisation of a novel interactive electronic medication safety dashboard in general practice: a mixed methods study. *BMC Medical Informatics and Decision Making,* 2020;20(69)

23. Swinglehurst D, Greenhalgh T, Russell J, *et al.* Receptionist input to quality and safety in repeat prescribing in UK general practice: ethnographic case study. *BMJ.* 2011;343:d6788.

24. Slight SP, Howard R, Ghaleb M, *et al.* The cause of prescribing errors in English general practices: a qualitative study. *British Journal of General Practice* 2013;63:e713-e720

25. Scott IA, Pillans PI, Barra M, *et al.* Using EMR-enabled computerized decision support systems to reduce prescribing of potentially inappropriate medications : a narrative review. *Therapeutic Advances in Drug Safety* 2018;9(9):559-573.

26. Silsand L, Ellingsen G. Complex decision making in clinical practice .Computer-Supported Cooperative Work February 27th-March 2nd 2016, San Francisco CA, USA.

27. Jia P, Zhang L, Chen J, *et al.* The effects of Clinical Decision Support Systems on Medication Safety: An Overview. *PLoS ONE* 2016;11(12):e0167683.

28. Nanji KC, Slight SP, Seger DL, *et al.* Overrides of medication-related clinical decision support alerts in outpatients. *JAMA* 2014;21:487-491.

29. Avery AJ, Savelyich BSP, Sheikh A, *et al.* Identifying and establishing consensus on the most important safety features of GP computer systems: e-Delphi study. *Informatics in Primary Care,* 2005;13(1)3-12.

30. Hayward J, Thomson F, Milne H, *et al* “Too much, too late”: mixed methods multi-channel video recording study of computerized decision support systems and GP prescribing *Journal of the American Medical Informatics Association* 2013;20(e1):e76-e84.

For peer review only - http://bmjopen.bmj.com/site/about/guidelines.xhtml

31. Van der Sijs H, Aarts J, Van Gelder T, *et al.* Overriding of Drug Safety Alerts in Computerized Physician Order Entry. *JAMA* 2006;13:138–147.

32. Greenhalgh T, Wherton J, Papoutsi C, *et al*. Beyond adoption: A new framework for theorizing and evaluation nonadoption, abandonment and challenges to the scale -up, spread, and sustainability of health care technologies. *Journal of Medical Internet Research,* 2017;19(11):e367 p1.

33. Jeffries M, Keers RN, Phipps DL *et al.* Developing a learning health system: insights from a qualitative process evaluation of a pharmacist-led electronic audit and feedback intervention to improve medication safety in primary care. *PLoS ONE* 2018;13(10):e0205419

34.Mair F, May C, O'Donnell C, *et al.* Factors that promote or inhibit the implementation of e-health systems: an explanatory systematic review. *Bulletin of the World Health Organisation* 2012;90(3):57–364.

35. Jeffries M, Salema N, Laing L. *et al.* The Implementation, Use and Sustainability of a Clinical Decision Support System for Medication Optimisation in Primary Care: A Qualitative Evaluation *PLoS ONE* 2021;16(5): e0250946.

36. Shamsuddin A, Jeffries M, Keers RN, *et al.* Strategies for success of interventions to support prescribing safety in primary care. *BJGP Open.* 2021;5(5):

37. Cresswell KM, Sadler S, Rodgers S *et al.* An embedded longitudinal multi-faceted qualitative evaluation of a complex cluster randomized controlled trial aiming to reduce clinically important errors in medicines management in general practice. *Trials* 2012;13:78.

38. Braun V, Clark V. To saturate or not to saturate? Questioning data saturation as a useful concept for thematic analysis and sample-size rationales. *Qualitative Research in Sport, Exercise and Health* 2021;13(2):201-216.

For peer review only

39. Braun V, Clark V. Using thematic analysis in psychology. *Qualitative Research in Psychology* 2006;3(2):77-101.

40. King N. 'Doing template analysis', in Symon G, Cassell C (eds.) *Qualitative Organizational Research: Core Methods and Current Challenges.* London: Sage, 2012 pp256-70.

41. Hodkinson A, Tyler N, Ashcroft, DM, *et al.* Preventable medication harm across health care settings: a systematic review and meta-analysis. *BMC Medicine* 2020;18:313.

42. Bower P, Macdonald W, Harkeness E, *et al*. Multimorbidity, service organisation and clinical decision making in primary care : a qualitative study. *Family Practice* 2011;*28(5):* 579-87.

43. Moffat K, Mercer SW. (2015) Challenges of managing people with multimorbidity in today's healthcare systems. *BMC Family Practice* 2015;16(10):129.

44. Waring J, Allen D, Braithwaite J, *et al*. (2015) Healthcare quality and safety: a review of policy, practice and research. *Sociology of Health and Illness* 2015;38(2):198-215

45. Trinkley KE, Blakeslee WW, Matlock DD, *et al.* Clinician preferences for computerised clinical decision support medications in primary care: a focus group study. *BMJ Health Care Informa*tics 2019;26.

46.Long KM, McDermott F, Meadows GN. (2018) Being pragmatic about healthcare complexity: our experiences applying complexity theory and pragmatism to health services research. *BMC Medicine* 2018;*16*(94).

47. Baqir W, Barrett S, Desai N, *et al*. A clinic-ethical framework for multidisciplinary review of medication in nursing homes. *BMJ Quality Improvement Reports* 2016;u203261 w2538.

For peer review only - http://bmjopen.bmj.com/site/about/guidelines.xhtml

48. Baqir W, Hughes J, Jones T, *et al*. Impact of medication review, within a shared decision-making framework, on deprescribing in people living in care homes. *European Journal of Hospital Pharmacy* 2017;24:30-33.

.

.

Supporting Information:- Appendix A1 - Interview Schedule

For peer review only

THE UNIVERSITY of EDINBURGH

INTERVIEW SCHEDULE

Interviewer introduction:

I am [….] and I am a researcher at The University of Manchester. We are carrying out a project looking at how the clinical decision support (CDS) software tool Optimise Rx® is introduced and used over time in general practices. We are particularly interested in the role of this tool in the safe prescribing and monitoring of medications as it incorporates 'prescribing safety indicators' [describe them]. We would like to find out more about the experiences of primary care staff, managers and software developers who have worked with the Optimise Rx® system in some way.

This interview will last for up to one hour, but if this is not your first interview with us it may take more like 30 minutes. During our time together I'd like to discuss your experiences of Optimise Rx® in practice, with a focus on medication safety practices. If this is not your first interview, I would like to revisit some of the things you said last time for an update, whilst also exploring any new topics that you want to raise today. I should remind you that the interviews are confidential. I'd like to audio record the discussion if that is okay with you; this is simply to help me capture all of the information that comes out of it. If you prefer, I can make written notes instead. You can ask for the audio-recorder to be switched off at any point during the interview. The recordings will be destroyed as soon as they are transcribed, the transcripts will be kept in a secure location for five years after the study is complete and then they will be destroyed.

Before we begin, I'd like to provide some ground rules for the discussion:

- You are being digitally recorded, so speak clearly;
- We will anonymise the transcript so that nobody or nowhere can be identified by name. However, please try to avoid naming specific people or locations;
- Everything discussed here is confidential. However, if you were to reveal anything that would be considered unlawful or anything that would place you at somebody else at risk of harm, we may have to report this to a clinical supervisor or manager.

Unless you have any questions for me, then we can begin.

For peer review only - http://bmjopen.bmj.com/site/about/guidelines.xhtml

Part one: background

Can you tell me a little about your professional background and current place(s) of work?

- Name and gender
- Profession
- Years of experience
- Current job title and place of work

The CDS system Optimise Rx®

- (practice staff) how long has Optimise Rx® been installed, where is it installed
- Was anything like Optimise Rx® installed previously
- At what point did you become involved with the CDS system

For peer review only

For peer review only - http://bmjopen.bmj.com/site/about/guidelines.xhtml

Part two: main discussion

Questions chosen will depend upon how many interviews have been conducted with the participant and what past response(s) were, as multiple interviews may take place over time at 0-6 months, 6-18 months, and 18 months + post implementation of Optimise Rx®. Some members of staff may only be interviewed once or twice, at early or late time points.

1. *Can you describe how Optimise Rx® was introduced /continues to be used into your workplace?*
    a. Who drove things forward and how, did they involve others
    b. Roles – you and management
    c. Allocation of resources and training/support for the tool – who, what, when
    d. What went well, what could be done better

2. *What do you think Optimise Rx® is there for and how it should it be used? (changing views?)*
    a. Focus participant on prescribing and monitoring safety, and the indicators
    b. Do your colleagues share your views?
    c. Do some people know more about the tool than others? What is the impact of this
    d. Do you think that the tool has actual or potential value / benefits for your work?

3. *Please describe how Optimise Rx® is used / continues to be used by you and your colleagues as part of your work, in the context of prescribing and monitoring safety.*
    a. Where possible focus attention on prescribing safety indicators
    b. Initial views/expectations versus reality of use
    c. Have you contributed to the CDS system
    d. Frequency and nature of interaction with the tool over time
    e. Who uses the tool and what outputs are generated, are some users different

4. *Can you tell me about how Optimise Rx® 'fits in' with your existing work? (over time)*
    a. Where possible focus attention on prescribing safety indicators
    b. Has the tool changed the way you or the team works?
    c. Has the tool changed the nature of yours or others work – type, volume, order
    d. Have you or others adapted your work to suit the tool, or vice versa
    e. Has the tool become an integral part of daily work? How was this achieved
    f. Do you (still) think that using the tool should be part of your role/duties and why?
    g. Do you (still) think that this CDS system should be used routinely across primary care?

For peer review only - http://bmjopen.bmj.com/site/about/guidelines.xhtml

5. *How well does Optimise Rx® work in your practice(s)?*
    a. Is the information produced by the CDS in relation to prescribing and monitoring safety: understood, timely, relevant, useful?
    b. Have the potential benefits of the tool been realised?
    c. Are there any problems with how well the CDS system works and how are these dealt with, e.g. technical issues, interface issues
    d. Has the system disrupted working relationships?
    e. Is any information collected to find out how well the CDS system is working for you and the team? Who is responsible and how is this information used
    f. Do you have any ways in which the team get together to talk about the CDS tool and how well it is working for you? Who is responsible for driving/organising this and what have been the outputs of such activity
    g. What is your opinion of the value of the system on your work, and does this differ to the wider team?
    h. Do you have confidence in the system – what gives you confidence or takes it away
    i. Do you have confidence in others using the system, are some people more skilled than others in using it and how does this affect things
    j. Example of where the CDS has improved / struggled to improve care?
    k. Do you support the use / ongoing use of the system?

Interviewer conclusion:

Concluding questions

- *Is there anything that you would like to talk about?*
- *Is there anything that you would like to go back and talk about?*

Switch off tape recorder

[INTERVIEWER TO ARRANGE SUBSEQUENT INTERVIEW(S) IF REQUIRED AND PRACTICAL]

Many thanks for taking the time to help us with this study. Your contribution has been extremely valuable. If you wish we can send you a copy of your interview transcript, and you can also request to receive a summary of the findings of this research study, just ask us. In the meantime please feel free to contact either myself or the other researcher(s) involved if you have questions in future.

For peer review only - http://bmjopen.bmj.com/site/about/guidelines.xhtml

Using Sociotechnical Theory to Understand Medication Safety Work in Primary Care and Prescribers use of Clinical Decision Support: A Qualitative Study.

COREQ checklist

Note: in order to minimize the length of the manuscript, some of the details on the checklist (marked '*') are not included in the manuscript.

Guide question		*Response*	*Page number in manuscript*
1	Interviewer/facilitator	MJ conducted the interviews.	P9
2	Credentials	MJ holds an PhD in Pharmacy Practice	*
3	Occupation	MJ : Research Associate in medication safety;	*
4	Gender	MJ male	*
5	Experience and training	MJ has previous experience of undertaking qualitative research in healthcare at PhD and postdoctoral level	*
6	Relationship established	The researchers were not known to the participants prior to the study	*
7	Participant knowledge of the researcher	Participants were made aware of the reasons for doing the research via the information which was given to the participant prior to the interview	Sampling and recruitment P8; Ethics statement
8	Researcher characteristics	The researchers had identified the study topic as part of larger programmes of work in their research groups, medication safety in primary care.	*
9	Methodological orientation and theory	Sociotechnical theory	Background
10	Sampling	The sampling was purposive and included people involved in prescribing medicines who had knowledge and experience of using CDS in their daily work in general practices within the four CCG areas, including GPs, general practice nurses and general practice-based pharmacists. Sampling and recruitment of general practices was facilitated through discussions with CCG managers. MJ approached potential practices between December 2017 and November 2019, by email or telephone and invited them to take part. In addition MJ visited CCG meetings of groups of practices. After each practice had indicated that their staff might be interested in taking part, the practice was visited by MJ to further explain the study and provide written information. Practices consented to take part in the study before individual staff were approached. Individual general practice staff were approached directly by telephone or email, or through liaising with the general practice manager. A total of 41 practices were approached; nine practices consented to take part and 32 either declined to take part or did not respond.. Practices commonly gave time and staffing	P8

		commitments as reasons for not taking part. Each potential participant was provided with written information about the study and given a minimum of 24 hours to decide if they wished to take part. MJ then contacted those staff wanting to take part by telephone or email to arrange a convenient time for the interview. The interview process was two stage with follow-up interviews conducted approximately 12 months after the first interview with a convenience sample of different participants (n=7) in order to understand any changes that may have occurred and if any new themes were emerging.	
11	Method of approach	Participants were approached by telephone or email	P8-9
12	Sample size	Health professionals at 41 general practices	P8
13	Non-participation	A number of possible participants were approached but declined to participate. This was predominantly this was for reasons of time, workload or lack of use of the system.	*
14	Setting of data collection	All interviews were conducted by MJ and took place at the participants' usual place of work (general practice, CCG offices)	P9
15	Presence of non-participants	No non-participants were present	*
16	Description of sample	Twenty-three interviews were conducted with 20 participants (14 GPs, 3 nurse prescribers and 3 practice pharmacists). The first interviews were conducted between February 2018 and November 2019 with follow-up interviews conducted between August 2019 and June 2020. Interviews ranged in duration from 13-41 minutes with a mean length of 31 minutes.	P9
17	Interview guide	The semi-structured interview schedule was informed by previous studies in medication safety	P7 Appendix S1
18	Repeat interviews	7 follow-up interviews were conducted	P8
19	Audio/visual recording	Audio recording only, with consent from the participant	Ethics statement
20	Field notes	None	*
21	Duration	Interviews ranged in duration from 13-41 minutes with a mean length of 31 minutes.	P9
22	Data saturation	Data collection continued until saturation was reached and no new themes emerged from the interviews..	P9
23	Transcripts returned	No transcripts were returned to participants	*
24	Number of data coders	MJ coded the data but regular discussions codes were held with co-authors	P9-10
25	Description of the coding tree	A coding tree description is not given. Coding themes were developed from the first transcripts and then developed through discussions amongst the authors	P9-10
26	Derivation of themes	Emerging thematic codes were applied to the data and new themes emerged from the data. This is described in the analysis section	P9-10
27	Software	QSRNvivo 12 software was utilised to manage the data	P9
28	Participant checking	No	*
29	Quotations presented	Please see the results section of the manuscript	P10-P16
30	Data and findings consistent		P10-P16
31	Clarity of major themes		P10-P16
32	Clarity of minor themes		P10-P16

For peer review only

BMJ Open

Using sociotechnical theory to understand medication safety work in primary care and prescribers' use of clinical decision support: A qualitative study.

Journal:	*BMJ Open*
Manuscript ID	bmjopen-2022-068798.R1
Article Type:	Original research
Date Submitted by the Author:	06-Mar-2023
Complete List of Authors:	Jeffries, Mark; The University of Manchester, NIHR Greater Manchester Primary Care Patient Safety Translational Research Centre; The University of Manchester, Centre for Pharmacoepidemiology and Drug Safety, Division of Pharmacy and Optometry, School of Health Sciences<br>Salema, Nde-Eshimuni; University of Nottingham, Centre for Academic Primary Care; The University of Manchester, NIHR Greater Manchester Primary Care Patient Safety Translational Research Centre.<br>Laing, Libby; University of Nottingham, Centre for Academic Primary Care<br>Shamsuddin, Azwa; University of Hull, School of Health Sciences<br>Sheikh, Aziz; University of Edinburgh, Division of Community Health Sciences<br>Avery, Tony; University of Nottingham, Primary Care<br>Chuter, Antony; University of Nottingham<br>Waring, Justin ; University of Birmingham<br>Keers, Richard; University of Manchester, Centre for Pharmacoepidemiology and Drug Safety ; Suicide, Risk and Safety Research Unit, Greater Manchester Mental Health NHS Foundation Trust, Manchester
**Primary Subject Heading**:	Health services research
Secondary Subject Heading:	General practice / Family practice, Qualitative research, Health informatics
Keywords:	Quality in health care < HEALTH SERVICES ADMINISTRATION & MANAGEMENT, Primary Care < Primary Health Care, QUALITATIVE RESEARCH

SCHOLARONE™
Manuscripts

I, the Submitting Author has the right to grant and does grant on behalf of all authors of the Work (as defined in the below author licence), an exclusive licence and/or a non-exclusive licence for contributions from authors who are: i) UK Crown employees; ii) where BMJ has agreed a CC-BY licence shall apply, and/or iii) in accordance with the terms applicable for US Federal Government officers or employees acting as part of their official duties; on a worldwide, perpetual, irrevocable, royalty-free basis to BMJ Publishing Group Ltd ("BMJ") its licensees and where the relevant Journal is co-owned by BMJ to the co-owners of the Journal, to publish the Work in this journal and any other BMJ products and to exploit all rights, as set out in our licence.

The Submitting Author accepts and understands that any supply made under these terms is made by BMJ to the Submitting Author unless you are acting as an employee on behalf of your employer or a postgraduate student of an affiliated institution which is paying any applicable article publishing charge ("APC") for Open Access articles. Where the Submitting Author wishes to make the Work available on an Open Access basis (and intends to pay the relevant APC), the terms of reuse of such Open Access shall be governed by a Creative Commons licence – details of these licences and which Creative Commons licence will apply to this Work are set out in our licence referred to above.

Other than as permitted in any relevant BMJ Author's Self Archiving Policies, I confirm this Work has not been accepted for publication elsewhere, is not being considered for publication elsewhere and does not duplicate material already published. I confirm all authors consent to publication of this Work and authorise the granting of this licence.

For peer review only

Using sociotechnical theory to understand medication safety work in primary care and prescribers' use of clinical decision support: A qualitative study.

Mark Jeffries (Corresponding Author)

NIHR Greater Manchester Patient Safety Translational Research Centre, Division of Pharmacy and Optometry, School of Health Sciences, Room 1.134 | Stopford Building | The University of Manchester | Oxford Road | Manchester | M13 9PT | 0161 275 3680
mark.jefferies@manchester.ac.uk

Nde-Eshimuni Salema [1, 2]

Libby Laing [1,3],

Azwa Shamsuddin [3,4]

Aziz Sheikh,[4]

Anthony J Avery[1, 2]

Antony Chuter [5]

Justin Waring[7]

Richard N Keers [1,7,8]

[1] NIHR Greater Manchester Primary Care Patient Safety Translational Research Centre, University of Manchester, Manchester Academic Health Sciences Centre (MAHSC), Manchester, United Kingdom.

[2] Centre for Academic Primary Care, School of Medicine, University of Nottingham, Nottingham, United Kingdom.

[3] School of Health Sciences, Cottingham Road University of Hull, United Kingdom.

[4]Usher Institute, University of Edinburgh, United Kingdom.

[5] 68 Brighton Cottages, Copyhold Lane, Lindfield, Haywards Heath, West Sussex, United Kingdom.

For peer review only

[6]School of Social Policy, Health Services Management Centre, University of Birmingham, United Kingdom.

[7] Centre for Pharmacoepidemiology and Drug Safety, Division of Pharmacy and Optometry, School of Health Sciences, University of Manchester, Manchester, United Kingdom. 0161 275 3680

[8] Suicide, Risk and Safety Research Unit, Greater Manchester Mental Health NHS Foundation Trust, Manchester, United Kingdom.

Word Count 5370

For peer review only

ABSTRACT

Objectives

The concept of safety work draws attention to the intentional work of ensuring safety within care systems. Clinical Decision Support (CDS) has been designed to enhance medication safety in primary care by providing decision-making support to prescribers. Sociotechnical theory understands that healthcare settings are complex and dynamically connected systems of fluid networks, human agents, changing relationships and social processes. This study aimed to understand the relationship between safety work and the use of CDS.

Design and setting

This qualitative study took place across nine different general practices in England. Stakeholders included general practitioners (GPs) and general practice-based pharmacists and nurse prescribers. Semi-structured interviews were conducted to illicit how the system was used by the participants in the context of medication safety work. Data analysis, conducted alongside data collection was thematic, and drew upon socio-technical theory

Participants

Twenty-three interviews were conducted with 14 GPs, three nurse prescribers and three practice pharmacists between February 2018 and June 2020.

Results

Safety work was contextually situated in a complex network of relationships. Three interconnected themes were interpreted from the data: i) the use of CDS within organisational and social practices and workflows; ii) safety work and the use of CDS within the interplay

For peer review only

between prescribers, patients and populations; and iii) the affordances embedded in CDS systems.

Conclusion

The use of sociotechnical theory here extends current thinking in patient safety particularly in the ways that safety work was co-constituted with the use of CDS alerts. This has implications for implementation and use to ensure that the contexts into which such clinical decision support systems are implemented are taken into account. Understanding how alerts can adapt safety culture will help improve the efficacy of CDS systems, enhance prescribing safety and help to further understand how safety work is achieved in primary care.

Strengths and limitations of this study

- The use of sociotechnical theory, to frame the thematic analysis enabled a nuanced understanding of the ways in which the CDS was utilised for medication safety work.
- This study had a longitudinal design with a variety of stakeholders from different health professions in primary care interviewed over time across multiple geographical locations.
- Semi-structured interviews were conducted with a full range of different prescribers in different roles (GPs, nurses and pharmacists).

For peer review only

For peer review only - http://bmjopen.bmj.com/site/about/guidelines.xhtml

INTRODUCTION

Healthcare services are increasingly interpreted as complex systems comprising fluid networks of people and technologies that interact in dynamic, non-linear and inter-dependent ways. [1-4] New thinking in the field of patient safety, often termed Safety-II, that focuses on 'work-as-done', rather than 'work-as-imagined', understands that safety is achieved less because people follow strict rules but rather because people adapt and respond to unanticipated events.[3 5] This idea departs from the 'measure and manage' orthodoxy of patient safety policies by attending to the situated practices of care work in the context of wider and inherently complex socio-cultural systems. [6 7] The concept of 'safety work' captures the idea that clinicians routinely need to engage in purposeful activities to actively create and restore safety in the context of dynamic and changeable systems.[8] Safety work has been understood from the concept of the multidimensional and dynamic safety culture of primary healthcare organisations. [9] This offers a way of exploring the interplay between system factors and also the purposeful behaviours of clinicians that goes beyond the reductionist and linear view of established safety perspectives, and instead foregrounds the combined influence of context and work as shaping clinical safety. Safety work has been seen to entail an understanding of safety culture amongst staff [9,] systems and collaboration between reception, administration and medical staff [10],articulation work [11] development of relationships and engagement with stakeholders [12 13] and management of workload. [8]

Sociotechnical theory, that draws upon interdependnet and constructionist theories considers that people and technology are reciprocally and recursively related and as a consequence the outcomes of the relationships between the social, human agency and technology are considered as interdependent and not simply as the interactions between homogenous unique elements. [14, 15] This approach focuses upon the social processes involved in the use of the technology since it sees technological use and adoption as a social practice that may involve

For peer review only - http://bmjopen.bmj.com/site/about/guidelines.xhtml

negotiation and conflict.Interdependent sociotechnical theories do not understand technology as something static that is external to social contexts but as part of that context. [16 17.] The implementation of Information Technology (IT) in health care settings has been explored from such sociotechnical perspectives.. [18 19] IT does not operate in isolation from social and organisational contexts and as such plays a potential role in both adding to system complexity and hence unsafe practice, and also contributing to the safety work of people within these systems. [1 20]

Extending this view, sociomateriality is a combination of the material properties of the technology with social processes. Sociomateriality considers that properties of the technology may be fixed therefore allowing for differences in use to be assigned to such social processes and contexts. [17] Technology has been conceptualised as offering affordances in which the material properties of the technology, through interaction with human agents, allow for the possibilities for certain actions. [21-23] This contrasts to approaches that might focus narrowly and deterministically upon the functions and capacities of the technology. In the concept of affordances , technology has the possibility of both shaping human action and being shaped by different social processes and practices. [21] New rules and conventions may evolve in a dynamic interaction between the technology, users and contexts that then changes social processes and practices. [16 17]In this way technology is not a casual facilitator but affords the possibility of action as one element of a complex social process. [17, 21, 22]

Medications safety technologies

The World Health Organization's (WHO) Third Global Patient Safety Challenge: Medication Without Harm places medication safety as a global priority [24] Prescribing errors in general practice are an important and expensive cause of preventable safety incidents, illness, hospitalisations and deaths. [25] Medication safety across primary care operates within complex contexts that often involves collaboration between different general practice staff, between

For peer review only - http://bmjopen.bmj.com/site/about/guidelines.xhtml

people and technology and is a fluid, negotiated, social process. [10 26 27]Prescribing errors have been considered to occur because of a multitude of error producing conditions including prescriber characteristics, patient characteristics, the working environment, the specific tasks and issues with technology. [28]

Prescribing safety in general practice is increasingly promoted through the use of specialist Clinical Decision Support (CDS) tools used to provide clinicians with clinical knowledge and patient related information to improve the quality of prescribing and reduce medication error. [29 30] The CDS system evaluated here has been in use across general practices in England and Wales since 2014. The CDS is embedded within the GP clinical system and provides a range of patient specific messages that appear at the point of prescribing as a dialogue box or 'pop-up'. Prescribers have full discretion as to whether they accept the message. Previous evaluation of CDS systems focused upon those designed to improve care outcomes often in hospital settings. [29 31 32] CDS has been seen as moderately successful, but it is widely acknowledged that prescribers often override alerts due to high volumes and limited perceived value ('alert fatigue'), and particularly if they interrupt clinical workflow by arriving at inappropriate times in the consultation.[32-35] 'Success' or 'failure' in implementation of IT has in the past been attributed to material properties of the technology and interoperability, user characteristics, and organisational settings and processes. [36] It has however been suggested that discrete individual barriers and facilitators are less important than the complex interactions between those factors. [16 36]Engagement between the health professionals working in clinical settings and the technology may also inform understanding of the potential impact on changes in working practices, roles and responsibilities. [12 37] There has been little in the way of qualitative research that has utilised sociotechnical theory to understand how medication safety alerts might operate as part of the safety work undertaken by those working within primary care. This study aimed to apply sociotechnical theory to

For peer review only - http://bmjopen.bmj.com/site/about/guidelines.xhtml

understand the use of CDS in the accomplishment of safety work in primary care, to understand how CDS transforms safety work, and how new forms of safety work are involved in the implementation and use of CDS.

METHODS

Study design

This qualitative study used semi-structured interviews with multiple stakeholders working as prescribers in general practices in England. The study took place across nine different general practices within four separate Clinical Commissioning Groups (CCGs), in the North West and East Midlands regions of England. The study was part of a wider qualitative longitudinal process evaluation of interventions designed to improve prescribing safety in primary care.[38,39]

Sampling and recruitment of participants

The sampling was purposive and included people involved in prescribing medicines who had knowledge and experience of using CDS in their daily work in general practices within the four CCG areas, including GPs, general practice nurses and general practice-based pharmacists. Sampling and recruitment of general practices was facilitated through discussions with CCG managers. MJ approached potential practices between December 2017 and November 2019, by email or telephone and invited them to take part. In addition MJ visited CCG meetings of groups of practices. After each practice had indicated that their staff might be interested in taking part, the practice was visited by MJ to further explain the study and provide written information. Practices consented to take part in the study before individual staff were approached. Individual general practice staff were approached directly by telephone or email, or through liaising with the general practice manager. A total of 41 practices were approached; nine practices consented to take part and 32 either declined to

For peer review only - http://bmjopen.bmj.com/site/about/guidelines.xhtml

take part or did not respond. Practices commonly gave time and staffing commitments as reasons for not taking part. Each potential participant was provided with written information about the study and given a minimum of 24 hours to decide if they wished to take part. MJ then contacted those staff wanting to take part by telephone or email to arrange a convenient time for the interview. The interview process was two stage with follow-up interviews conducted approximately 12 months after the first interview with a convenience sample of different participants (n=7) in order to understand any changes that may have occurred and if any new themes were emerging.

Data collection

The semi-structured interview schedule was developed by MJ and RK drawing upon previous studies in medication safety in primary care. [12 19 40] This was designed to illicit how CDS was used by the participants in the context of medication safety work (see Appendix S1). In order to ensure it remained appropriate the interview schedule was reviewed throughout data collection. Interviews continued alongside data analysis in an iterative approach until data saturation was reached. We understood data-saturation as being achieved when we judged further interpretation of codes and themes would not provide further insights. [41]All interviews were conducted by MJ and took place face-to-face at the participants' usual place of work (general practice, n=19) or by telephone (n=4); all were digitally audio-recorded. Participants were offered a £20 shopping voucher per interview as acknowledgement of their time. Twenty-three interviews were conducted with 20 participants (14 GPs, 3 nurse prescribers and 3 practice pharmacists). Follow-up interviews were conducted with 7 participants (5 GPs, 1 nurse, 1 practice pharmacist).The first interviews were conducted between February 2018 and November 2019 with follow-up interviews conducted between August 2019 and June 2020. Interviews ranged in duration from 13-41 minutes with a mean length of 31 minutes.

Data analysis

For peer review only - http://bmjopen.bmj.com/site/about/guidelines.xhtml

Data analysis was informed by interdependent and constructionist sociotechnical theories and models. [14 15 16] MJ led the analysis, which was thematic and conducted alongside data collection following verbatim transcription. The analysis followed two parts: firstly, an inductive thematic approach informed by Braun and Clarke [42] and secondly a deductive template approach which involved developing a coding template from the first stage. [43] For the initial inductive thematic approach, MJ used QSR NVIVO 12 to organise the data and inductively coded early interviews to identify features, patterns, groups of codes and potential themes to build a preliminary framework for application to the data and to inform further data collection. These codes were discussed, in the context of sociotechnical theory, with the wider research team (RK NS LL AC ASha). These discussions led to the development of a further coding template that was applied to the full data set, refined and reworked. From this coding, the final themes as presented in the results were interpreted.

Patient and Public involvement

AC is a patient and public representative who was involved in all aspects of the study and is a co-author of the paper.

RESULTS

Interpretative data analysis found a complex network of relationships between the technology, different users and the contexts in which the technology was utilised. Safety work and the use of CDS was contextually situated in this network and reciprocally interrelated with it.

Three broad, interconnected themes were interpreted from the interview data.

- The use of CDS within organisational and social practices and workflows;
- The use of CDS within the interplay between prescribers, patients and populations;

For peer review only

- Sociomateriality – the affordances embedded in CDS.

The use of CDS within organisational and social practices and workflows

For many prescribers the use of CDS reflected their established commitment to promoting safety within their practice which was described as illustrative of their a *'safety climate'*.

> *I think we are a safety conscious practice […] we don't want mistakes to happen so therefore creating a safe climate where you feel you are doing things safety is actually really important to us as a practice* ***GP12***

Organisational culture varied. One GP reflected upon these variations across different sites of the group practice in which they worked. One site had a strong focus upon being *'traditionally good prescribers'* who had '*work(ed) very hard […] on the prescribing* ***(GP3)****.* Such 'good' prescribing was seen to be about stability which (**GP3**) suggested '*creates much improved prescribing'* in contrast to another site of their group practice which, they said, had high staff turnover. This was reflected in the number of CDS alerts seen. Having a '*safety system'* was a way of avoiding many alerts. A safety culture that included medication reviews and which had been '*done …for so long that it's relatively slick',* was perceived as conducive to utilising alerts from CDS systems in a way that avoided being overwhelmed.

> *Medication reviews I do here. This probably might explain why we've got less (alerts) here. We do a six month or annual review on people that are only on two things here, and we've done it for so long that it's relatively slick. Whereas other places where they've not had review I'm going into a jungle almost of interactions and possible problems. They've never had any safety system before, so there's lots of alerts will go on.* ***GP3***

Organisational practices involved workflows where the use of the system was most pertinent in medication reviews. The following GP utilised CDS mostly when undertaking repeat

For peer review only - http://bmjopen.bmj.com/site/about/guidelines.xhtml

prescribing. This was perceived to lead to disregarding alerts because '*the patient's not in front of you'* and because of the volume of prescriptions that needed repeat authorisation.

> *I don't often use it (CDS support) in consultations, because I suppose that's one of the problems of it, […] the time it crops up in workflows most, is when you're signing today's 60 repeat prescriptions, so the patient's not in front of you, but there's a medication review overdue, […] (the alert) flashes up and tells you that something minor could need changing, and you sort of let it go.* ***GP13***

The use of CDS alerts within the interplay between prescribers, patients and populations.

The characteristics of patients and roles of prescribers impacted upon the CDS use for medication safety. Users with differing roles in general practice might not use CDS in the same way. The wider demographics of patient groups and broader holistic needs of individual patients were considered relevant to how prescribing would be prioritised and how CDS was used. For one GP, changes to specific individual medications, as alerted by CDS, was not patient's primary concern.

> *…if you've got a patient with 20 different drugs, […] they've just lost their job, they're about to become homeless, they've got epilepsy, depression, anxiety, diabetes that's out of control, they've got 15 appointments, but they've missed the last ten, and they've got a safeguarding issue with their child […] There are much, much bigger issues for them.* ***GP5***

Individual patient needs, particularly for patients with complex medical problems or multi-morbidity, could impact upon the ways in which CDS alerts were responded to by prescribers. This GP suggested that it was difficult to respond to alerts if patients were '*on long lists of medication already'* ***GP14***

For peer review only - http://bmjopen.bmj.com/site/about/guidelines.xhtml

Alerts were rejected if the prescriber disagreed and were considered inappropriate to the patient because of other morbidity, frailty or their social situation. Safety work was accomplished by the prescribers balancing alerts against their own knowledge and understanding of individual patient circumstances. In this specific case, leaving a patient with epilepsy without medication as the alert had suggested was perceived as less safe.

> *Sometimes it's hazardous not to prescribe [...] some of the epilepsy drugs you can't give them safely because they're dangerous drugs; and it's telling you all these warnings, but you've got to give something because it would be more dangerous to leave somebody fitting […] somebody with bad COPD (chronic obstructive pulmonary disease) and they're on a host of other drugs, there really isn't an antibiotic that you can give that doesn't have a risk of cardiac arrhythmia or something like that; (but) , I can't leave them without the antibiotic [...] we've got to give something.* ***GP1***

Safety work was also accomplished by different prescribers within the context of responsibilities, and moral and ethical behaviour. Prescribers reflected how alerts did not remove the responsibility of a prescribers signature and prescribers still had to justify their actions.

> *I don't think any one tool […] , you shouldn't rely on just one thing at all. You are still responsible for prescribing and what you prescribe so you've still got to justify it. You can overrule …* ***GP5***

Alerts were also seen within the context of balancing different responsibilities towards the patient, the wider practice population and the management of resources.

> *so our prime responsibility is to the patient who's in front of us now. But, at the same time, we have a responsibility to our whole practice population, and to the country, of managing resources correctly. So, we have three different responsibilities that exist,*

For peer review only - http://bmjopen.bmj.com/site/about/guidelines.xhtml

almost like a holy trinity, they're all there at the same time [...] And so anything that allows you to focus on one, while monitoring your progress on the other, is likely to be helpful, or feels helpful. So, you know, if I have someone in front of me who needs an antidepressant, and the current evidence is that sertraline is better than citalopram but I didn't get to see that, if (CDS) comes up and says, actually we're now supposed to be giving sertraline, I think, oh that's great, 'cause that's a helpful thing, 'cause it helps me monitor my other obligations at the same time. ***GP18***

Knowing that there was patient information within alerts meant that the clinician had a moral obligation to act upon that information.

....there's a degree of responsibility, if you know that you have a tool and, you know, and there's potentially a person sat there, who could have been looked at, who hasn't been looked at, and is subject to potential harm. You've got responsibility, haven't you, as a clinician, because that information is there for you to access. ***GP15***

Safety work and prescribing was undertaken differently by different prescribers. This nurse considered that whilst they had been prescribing for 11 years it was generally felt amongst nursing staff that prescribing was an "*extended role*". This led to a lack of confidence, a fear of making mistakes and the seeking of support..

I think the way nurses think because we see it as an extended role, it's not part of this. I think there's that element of it and we're always worried, we're always covering our back all the time. A doctor said to me once that it's the mind set of how we're trained, nurses are trained to question, have I done something wrong [...]it's how we think, scared to do something wrong. ***Nurse 1***

For peer review only - http://bmjopen.bmj.com/site/about/guidelines.xhtml

In contrast, the use of CDS was seen as being dependent upon experience and expertise. This GP suggested that it was easy to use and decide whether to accept alerts or not because they had many years' experience and was the prescribing lead for the practice. They suggested that they were more likely to rely upon their own experience and knowledge than less experienced prescribers who might be more likely to rely upon alerts.

Having been a GP for a long time, I'm also the prescribing lead for the practice [...], obviously, that relies on your own experience, skills, confidence, that sort of thing. I think probably as maybe one of the relatively older GPs, [...] having a reasonable amount of experience and confidence in what you're doing, probably means that you cancel it off more often than a younger one. ***GP14***

For this GP there was a clear contrast between how they undertook safety work using CDS and how that was conducted by their practice pharmacist. Practice pharmacists reported that they saw patients for longer appointment times and would be more likely to be dealing with medication reviews for patients with long-term conditions. Their contrasting work practices were related to differences in response to CDS alerts with the GP less likely to be methodical and more likely to not change medications in case the patient complained.

...it's been interesting seeing how our pharmacist works, now that (they are) seeing patients and doing (their) own medication reviews. (They are) much more thorough and [...] will challenge people a lot more and say, look you've been on this thing, I'm not sure you necessarily need that or you may be need something slightly different, and will go through it in a more methodical fashion. I think we do probably in our consultations, sort of, go through things a little bit more quickly. ***GP4***

Sociomateriality – the affordances embedded in the CDS.

For peer review only - http://bmjopen.bmj.com/site/about/guidelines.xhtml

CDS was used within Electronic Health Record (EHR) systems and was dynamically linked to other databases and clinical systems that could provide alerts. As a consequence this impacted upon how CDS was used, how users interacted with it, and how the alerts were responded to. Part of this was associated with a frustration about what different technologies could or could not do, or what could or could not be done with different technologies. Prescribers talked of CDS when embedded with one particular clinical system *'failing to launch' …'it's just so many more clicks on particular things of your screen'* ***GP12*** or '*the number of warnings that comes up in (name of clinical system) is far too many'* ***GP13.*** This was said to mean *'it slows down and frustrates you as you're doing an important task but at the same time you're not getting the advice from it as well.'* ***GP13***. Affordance in this way was not just about non-use or abandonment but about how that made users perceive the system and feel about using it, which then in turn led to a negative response. Such frustration with the CDS could lead to important medication safety issues.

> *We had a significant event where a medication was prescribed to a patient that shouldn't have been. We know that at least one of the factors in that was the fact that in the set up process of (the clinical system) different doctors chose different security settings for medicines safety….* ***GP13***

The perception that CDS was not providing accurate and useful alerts could mean that the system was ignored and therefore safety work potentially compromised.

In contrast, some participants described how CDS provided affordances for prescribers to undertake safety work. This GP considered the alerts not intrusive, easily overridden and providing useful and sensible safety advice. Such safety advice was seen as important since it could mitigate against safety problems for patients in the future.

For peer review only - http://bmjopen.bmj.com/site/about/guidelines.xhtml

I don't find them too intrusive really. I mean it is another thing that happens that didn't happen before but it's as we discussed before, it was they are relatively easy to override and the suggestions that are being made generally feel quite sensible [...] And if it's a safety thing, then generally I've been quite pleased that it's happened because it's given me an opportunity to make a change before potentially something that might cause me a problem later down the line ***GP4 (Follow-up interview)***

CDS was seen as providing prompts to safer prescribing that challenged prescribers to think about what they were about to prescribe. This could take the form, as described by this GP of avoiding a prescribing error.

... today it's prompted me not to prescribe the wrong dose of oxycodone. So off a picking list I was close to picking the wrong strengths of oxycodone. And that came up with an (CDS) prompt which was great. [...] , so I was very happy to have something held up in front of my face going, are you sure about that?. ***GP4***

DISCUSSION

The use of CDS for medication safety in primary care was seen to be contextually situated in the social practices of safety work. This builds upon understandings of healthcare settings as complex systems with different characteristics including people, tasks and technology. [3] In this study, safety work was accomplished within safety cultures, which varied for patients and prescribers and therefore shaped their particular forms of engagement with the CDS and safety work. The use of sociotechnical theory here extends current thinking in patient safety particularly in the ways that safety work was co-constituted with the use of CDS alerts.

Safety work in primary care

The safety work undertaken by prescribers when using CDS was often shaped by the perceived health needs and broader characteristics of the patient. Where CDS support was less easy to

For peer review only - http://bmjopen.bmj.com/site/about/guidelines.xhtml

accommodate to the holistic practices of prescribers, in terms of their relationship with patients, it was typically underutilised. Specifically, for patients with multiple and complex care needs, prescribers suggested it was necessary to consider factors other than prescribing, and relatedly the existing CDS system presented functional barriers to working with patients on multiple medicines. A recent systematic review and meta-analysis of medication harm across health settings found the highest incidence of medication harm in patients with high comorbidity and related polypharmacy. [44]It has previously been suggested that decision-making is made more complicated for clinicians when dealing with patients with multi-morbidity. [45 46] As such, the under-utilisation of CDS might be seen as stemming from a lack of appreciation amongst designers and policy-makers about how prescribing safety is shaped by the particular relationship between prescriber and patient, in the context of the former's prevailing safety practices and the latter's particular health needs.

We found that the utilisation of CDS for safety work within primary care was linked to the affordances within the technology and significantly how these related to the perceived needs of the patient and the associated approach to medical decision-making. That is, GPs' safety work often involved ignoring alerts where they were perceived as inaccurate, overwhelming, or irrelevant to the complexity of the decision-making for a given patient. Although our study did find that in other relational contexts, i.e. with less complex patients, or where there was more time available such as in consultations with practice pharmacists, CDS did provide prescribers with valued and useful alerts that were seen as enhancing their safety work.

CDS alerts were not neutral in that they provided a specific recommendation to the prescriber. This could be seen as form of 'digital nudging' whereby technology might be seen to influence choices and 'nudge' people into specific behaviours. [47] That the prescribers here did reject alerts suggests that this was an active rather than passive response to the alert CDS alerts afforded the possibility of making prescribing changes but were only acted upon if the

For peer review only - http://bmjopen.bmj.com/site/about/guidelines.xhtml

prescriber considered it appropriate within the given contexts of their practice. The system provided prompts and challenges in the form of alerts, which could be accepted, and acted upon in different ways, or ignored and thus there was a recursive relationship between people and the technology. [14]Responding to CDS alerts was therefore, not a linear and binary process but one that was fluid and contextually bound. Prescribers' responses to safety alerts were based upon their expertise and knowledge. Previously Swinglehurst [10] found that safety work in repeat prescribing was embedded in different types of organisational structures. This study reinforces the sense that primary care and medication safety operate as complex systems with multiple and interconnected parts. [1] We found that the organisational culture and what prescribers referred to as a 'safety climate' or 'safety culture' of particular importance with organisational practices such as workflows and tasks shaping the use of CDS. This reinforces that safety work is a social process operating within multidimensional and dynamic systems.[8 9 48] As well as organisational processes shaping the use of CDS, CDS alerts shaped the way prescribers accomplished safety work as they variously adapted their clinical practices and responded differently to them. [1 3] This included prescribers reflecting upon their moral and ethical responsibilities towards their patients and the wider community. This further highlights how safety work requires a flexible approach that is adaptive and responsive to different contextual situations and does not over rely upon technology. [10] Our study builds upon understandings of alert fatigue in that underutilisation cannot be therefore simply seen in terms of the volume and relevant of alerts but that alerts are part of the social process of managing safety within complex work in primary care.

Strengths and Limitations

A particular strength of this study is the focus upon sociotechnical theory, which enabled a nuanced understanding of the ways in which the CDS was utilised. By using this theoretical lens we could further understand how the CDS alerts and those already embedded in the EHR

For peer review only - http://bmjopen.bmj.com/site/about/guidelines.xhtml

were utilised within the context of organisational culture, and prescribers workflows. This builds upon the sociotechnical theory particularly that around the concept of affordances within technology that enable human actions.[21 22] Additionally, a further strength was the longitudinal design with a variety of stakeholders from different professions interviewed over time across multiple geographical locations. A full range of different prescribers in different roles (GPs, nurses and pharmacists) were interviewed but there could have been greater breadth across general practices. The present study found some variation between different prescribers (Pharmacists, GPs or Nurses) in the ways they adopted the CDS for safety work but we could only explore this in a limited way given our study sample. Further understanding how safety work is accomplished in the context of different users of the technology in future research would be useful. Many practice staff were also drawn from two of the four targeted CCG areas due to recruitment difficulties related to GP time and workload. Furthermore interviewing patients to gain their perspective may well have been useful. This study also relied solely upon interview data and may have benefited from observations or other ethnographic approaches that might have uncovered 'work-as-done'. [5]

Implications for future research, policy and practice.

CDS systems for medication safety have been utilised in primary care with varying success. [31 34 49] Since CDS systems operate within other technology such as the EHR it may be important to understand how different technologies interact and offer different ways people can engage in safety activities. Understanding open systems and the complexity in healthcare has been highlighted as important in health care evaluation. [1 50] Investment into CDS systems needs to be judged against how those systems will be used not just from a functionality aspect but also from the view of who the users will be and for which individual and groups of patients they will utilising the system for. How CDS are utilised for patients with multi-morbidity and polypharmacy will be an important consideration here. An important finding of our study was

For peer review only - http://bmjopen.bmj.com/site/about/guidelines.xhtml

that functionality was linked to patient characteristics and prescriber motivations in that where multiple alerts were received owing to patients having polypharmacy the system was then underutilised and the alerts could be ignored. Since this group of patients is at most risk from a prescribing error, this has important implications for practice. Multidisciplinary reviews of patients with polypharmacy have been found useful in other contexts including care homes. [51 52]

There is a recursive interrelationship between CDS alerts and safety culture in that CDS can enhance safety culture but may also be utilised differently within existing safety cultures as prescribers adapt their practices. [1 3] Further research could valuably explore how technology to enhance medication safety can shape organisational safety work and not just be utilised within a safety climate but be instrumental in building that safety culture. Understanding how alerts can adapt that safety culture and how prescribers may adapt to the alerts to form new emerging safety cultures is a challenge that will help improve the efficacy of CDS systems, enhance prescribing safety and help to further understand how safety work is achieved in primary care.

CONCLUSIONS

Sociotechnical theory enabled us to understand how the use of CDS for medication safety in primary care is context bound and dependent upon a complex and reciprocal network of relationships between the technology, different users and the places and spaces in which the technology was utilised. Where prescribers perceived their practices to have a safety culture this could impact upon the utilisation of CDS. The use of CDS for medication safety work was dependent upon who responded to alerts and for which patients the alerts were received. Since alerts for patients with multimorbidity and polypharmacy were reported to be underutilised an important implication and strategy of our findings is how prescribers may adapt their practices to ensure medication safety for this group of patients Prescribers adaptation of their practices in response to alerts has implications for the use of decision

For peer review only - http://bmjopen.bmj.com/site/about/guidelines.xhtml

support to ensure that the contexts into which it is implemented and therefore the ways in which safety work is accomplished are taken into account.

Contributors

AA secured funding. MJ, RK, AA and JW advanced the idea for the study. RK and AA provided study management. MJ collected the data. MJ led on data analysis with contributions from NS, LL, AC, RK, ASHE and ASHA. MJ drafted the manuscript which was reviewed and commented upon by all authors. All authors approved the final manuscript.

Funding

This study was part of the NIHR PRoTeCT Programme grant. PRoTeCT is funded by the NIHR Programme Grants for Applied Research Programme (1214-20012). The views expressed are those of the author(s) and not necessarily those of the NIHR or the Department of Health and Social Care.

Additionally MJ, RK, AA, LL and NS received funding from the National Institute for Health Research through the Greater Manchester Patient Safety Translational Research Centre (NIHR Greater Manchester PSTRC) grant number PSTRC-2016-003.

Competing interests The authors declare there is no conflict of interest.

Ethical approval and consent to participate

Ethical approval for the study was granted by the University of Manchester Research Ethics Committee. (Ref: 2017-0842-3977). Further approval to conduct the research was granted by the NHS Health Research Authority (IRAS project ID: 233079) and by the research and development offices for the NHS participating regions. General practices gave written informed consent for their staff to be approached to take part in the study. All interview participants gave written informed consent to take part in the study, and for interviews to be

For peer review only - http://bmjopen.bmj.com/site/about/guidelines.xhtml

audio-recorded, transcribed verbatim and for anonymised quotations to be used in publications.

Data availability statement

This is a qualitative study confined to relatively small groups of health care professionals in specific roles, particularly GP staff. Making the full transcripts publicly available could therefore potentially lead to the identification of participants. Our ethics approval was granted based on the anonymity of the individuals consenting to participate and specifically referred to only anonymised quotations being used in reports. As such the participants did not consent to full their transcript being made publicly available. Therefore, data cannot be made publicly available.

REFERENCES

1. Greenhalgh T Papoutsi C. Studying complexity in health services research: desperately seeking an overdue paradigm shift. *BMC Medicine* 2018;16:95.

2. Lipsitz LA. Understanding health care as a complex system. *JAMA* 2012;308(3):243-244.

3. McNab D, McKay J, Shorrock S, *et al.* Development and application of 'systems thinking' principles for quality improvement. *BMJ Open Quality* 2020;9e000714.

4. Pype P, Mertens F, Helewaut F, *et al.* Health care teams as complex adaptive systems: understanding team behaviour through team members perception of interpersonal interaction. *BMC Health Services Research* 2018;18:570.

5. Hollnagel E, Wears RL, Braithwaite J. From Safety-I to Safety-II: A White Paper. The Resilient Health Care Net. Published simultaneously by the University of Southern Denmark, University of Florida, USA, and Macquarie University, Australia 2015

For peer review only - http://bmjopen.bmj.com/site/about/guidelines.xhtml

6. Rowley E, Waring J. *A socio-cultural perspective on patient safety.* Boca Raton, FL, London, NewYork: CRC Press, 2017

7. Wears RL, Sutcliffe KM. *Still not Safe: Patient Safety and The Middle-Managing of American Medicine.* New York: OUP, 2020.

8. Daker-White G, Hays R, Blakeman T *et al*. Safety work and risk management as burdens of treatment in primary care : insights from a focused ethnographic study of patients with multimorbidity. *BMC Family Practice* 2018;19(155).

9. Kirk S, Parker D, Claridge T, *et al.* Patient safety culture in primary care: developing a theoretical framework for practical use *Quality and Safety in Health Care* 2007;16:313-320.

10. Swinglehurst D, Greenhalgh T, Russell J, *et al.* Receptionist input to quality and safety in repeat prescribing in UK general practice: ethnographic case study. *BMJ.* 2011;343:d6788.

11. Grant S, Mesman J, Guthrie B. Spatio-temporal elements of articulation work in the achievement of repeat prescribing safety in UK general practice. Sociology of Health & Illness 2016;38(2):306-324

12. Jeffries M, Keers RN, Phipps DL *et al.* Developing a learning health system: insights from a qualitative process evaluation of a pharmacist-led electronic audit and feedback intervention to improve medication safety in primary care. *PLoS ONE* 2018;13(10):e0205419

13. Jeffries M , Keers RN, Belither H, Sanders C, Gallacher K, Alqenae F, Ashcroft DM. Understanding the implementation, impact and sustainable use of an electronic pharmacy referral service at hospital discharge: A qualitative evaluation from a sociotechnical perspective. PLoS One. 2021 Dec 22;16(12):e0261153.

14. Orlikowski WJ, Scott SV. Sociomateriality: challenging the separation of technology, work and organization. *Academy of Management Annals* 2008;2(1):433-474.

For peer review only - http://bmjopen.bmj.com/site/about/guidelines.xhtml

15 Greenhalgh T, Shaw S, Wherton J *et al*. SCALS: a fourth generation study of assisted living technologies in their organisational, social, political and policy context. *BMJ Open.* 2016;6:e010208.

16. Klecun E. Transforming healthcare: Policy discourses of IT and patient-centred care. *European Journal of Information Systems* 2016;25:64-76.

17. Leonardi PM. 'Materiality, Sociomateriality and Sociotechnical systems: What do these terms mean? How are they different? Do we need them?' in Leonardi PM, Nardi BA, Kallinikos J. eds. *Materiality and Organizing: Social Interaction in a Technological World.* Oxford: OUP, 2012 pp.25-48.

18 Greenhalgh T, Stones R, Swinglehurst D. Choose and Book: a sociological analysis of 'resistance' to an expert system. *Social Science & Medicine* 2014;104:210-19.

19. Jeffries M, Phipps D, Howard RL, *et al.* Understanding the implementation and adoption of an information technology intervention to support medicine optimisation in primary care: qualitative study using strong structuration theory. *BMJ Open* 2017;7:e014810

20. Timmermans S, Berg M. The Practice of Medical Technology. *Sociology of Health and Illness,* 2003;25, 97-114.

21. Hutchby I. Technologies, texts and affordances. *Sociology* 2001;*35*(2):441-456.

22. Petrakaki D, Klecun E, Cornford T. Changes in healthcare professional work afforded by technology: The introduction of anational electronic patient record in an English hospital. *Organization* 2016;23(2):206-226.

23. Petrakaki, D, Waring J. Barber N. Technological affordances of risk and blame: the case of the electronic prescription service in England. *Sociology of Health and Illness* 2014;36(5):703-718.

For peer review only - http://bmjopen.bmj.com/site/about/guidelines.xhtml

24World Health Organization Medication Without Harm - Global Patient Safety Challenge on Medication Safety. Geneva: WHO, 2017

25 Elliott RA, Camacho E, Jankovic D, *et al.* Economic analysis of the prevalence and clinical and economic burden of medication error in England. *BMJ Quality & Safety* 2021;30:96–105.

26. Allen D, Braithwaite J, Sandall J *et al.* Towards a sociology of healthcare safety and quality. *Sociol. Health Illn*. 2016; 38(2):181-197.

27. Jeffries M, Gude WT, Keers RN, *et al.* Understanding the utilisation of a novel interactive electronic medication safety dashboard in general practice: a mixed methods study. *BMC Medical Informatics and Decision Making,* 2020;20(69)

28. Slight SP, Howard R, Ghaleb M, *et al.* The cause of prescribing errors in English general practices: a qualitative study. *British Journal of General Practice* 2013;63:e713-e720

29. Scott IA, Pillans PI, Barra M, *et al.* Using EMR-enabled computerized decision support systems to reduce prescribing of potentially inappropriate medications : a narrative review. *Therapeutic Advances in Drug Safety* 2018;9(9):559-573.

30. Silsand L, Ellingsen G. Complex decision making in clinical practice .Computer-Supported Cooperative Work February 27th-March 2nd 2016, San Francisco CA, USA.

31. Jia P, Zhang L, Chen J, *et al.* The effects of Clinical Decision Support Systems on Medication Safety: An Overview. *PLoS ONE* 2016;11(12):e0167683.

32. Nanji KC, Slight SP, Seger DL, *et al.* Overrides of medication-related clinical decision support alerts in outpatients. *JAMA* 2014;21:487-491.

For peer review only - http://bmjopen.bmj.com/site/about/guidelines.xhtml

33. Avery AJ, Savelyich BSP, Sheikh A, *et al.* Identifying and establishing consensus on the most important safety features of GP computer systems: e-Delphi study. *Informatics in Primary Care,* 2005;13(1)3-12.

34. Hayward J, Thomson F, Milne H, *et al* "Too much, too late": mixed methods multi-channel video recording study of computerized decision support systems and GP prescribing *Journal of the American Medical Informatics Association* 2013;20(e1):e76-e84.

35. Van der Sijs H, Aarts J, Van Gelder T, *et al.* Overriding of Drug Safety Alerts in Computerized Physician Order Entry. *JAMA* 2006;13:138–147.

36. Greenhalgh T, Wherton J, Papoutsi C, *et al*. Beyond adoption: A new framework for theorizing and evaluation nonadoption, abandonment and challenges to the scale -up, spread, and sustainability of health care technologies. *Journal of Medical Internet Research,* 2017;19(11):e367 p1.

37.Mair F, May C, O'Donnell C, *et al.* Factors that promote or inhibit the implementation of e-health systems: an explanatory systematic review. *Bulletin of the World Health Organisation* 2012;90(3):57–364.

38 Jeffries M, Salema N, Laing L. *et al.* The Implementation, Use and Sustainability of a Clinical Decision Support System for Medication Optimisation in Primary Care: A Qualitative Evaluation *PLoS ONE* 2021;16(5): e0250946.

39. Shamsuddin A, Jeffries M, Keers RN, *et al.* Strategies for success of interventions to support prescribing safety in primary care. *BJGP Open.* 2021;5(5):

40. Cresswell KM, Sadler S, Rodgers S *et al.* An embedded longitudinal multi-faceted qualitative evaluation of a complex cluster randomized controlled trial aiming to reduce clinically important errors in medicines management in general practice. *Trials* 2012;13:78.

For peer review only

41. Braun V, Clark V. To saturate or not to saturate? Questioning data saturation as a useful concept for thematic analysis and sample-size rationales. *Qualitative Research in Sport, Exercise and Health* 2021;13(2):201-216.

42. Braun V, Clark V. Using thematic analysis in psychology. *Qualitative Research in Psychology* 2006;3(2):77-101.

43 King N. 'Doing template analysis', in Symon G, Cassell C (eds.) *Qualitative Organizational Research: Core Methods and Current Challenges.* London: Sage, 2012 pp256-70.

44. Hodkinson A, Tyler N, Ashcroft, DM, *et al.* Preventable medication harm across health care settings: a systematic review and meta-analysis. *BMC Medicine* 2020;18:313.

45. Bower P, Macdonald W, Harkeness E, *et al*. Multimorbidity, service organisation and clinical decision making in primary care : a qualitative study. *Family Practice* 2011;*28(5):* 579-87.

46. Moffat K, Mercer SW. Challenges of managing people with multimorbidity in today's healthcare systems. *BMC Family Practice* 2015;16(10):129.

47 Weinmann, M, Schneider C, vom Brocke J. Digital nudging Business Information and Systems Engineering 2016;58(6):433-436

48Waring J, Allen D, Braithwaite J, *et al*. Healthcare quality and safety: a review of policy, practice and research. *Sociology of Health and Illness* 2015;38(2):198-215

49. Trinkley KE, Blakeslee WW, Matlock DD, *et al.* Clinician preferences for computerised clinical decision support medications in primary care: a focus group study. *BMJ Health Care Informa*tics 2019;26.

For peer review only - http://bmjopen.bmj.com/site/about/guidelines.xhtml

For peer review only

50.Long KM, McDermott F, Meadows GN. Being pragmatic about healthcare complexity: our experiences applying complexity theory and pragmatism to health services research. *BMC Medicine* 2018;*16*(94).

51. Baqir W, Barrett S, Desai N, *et al*. A clinic-ethical framework for multidisciplinary review of medication in nursing homes. *BMJ Quality Improvement Reports* 2016;u203261 w2538.

52. Baqir W, Hughes J, Jones T, *et al*. Impact of medication review, within a shared decision-making framework, on deprescribing in people living in care homes. *European Journal of Hospital Pharmacy* 2017;24:30-33.

Supporting Information:- Appendix A1 - Interview Schedule

For peer review only

INTERVIEW SCHEDULE

Interviewer introduction:

I am [....] and I am a researcher at The University of Manchester. We are carrying out a project looking at how the clinical decision support (CDS) software tool Optimise Rx® is introduced and used over time in general practices. We are particularly interested in the role of this tool in the safe prescribing and monitoring of medications as it incorporates 'prescribing safety indicators' [describe them]. We would like to find out more about the experiences of primary care staff, managers and software developers who have worked with the Optimise Rx® system in some way.

This interview will last for up to one hour, but if this is not your first interview with us it may take more like 30 minutes. During our time together I'd like to discuss your experiences of Optimise Rx® in practice, with a focus on medication safety practices. If this is not your first interview, I would like to revisit some of the things you said last time for an update, whilst also exploring any new topics that you want to raise today. I should remind you that the interviews are confidential. I'd like to audio record the discussion if that is okay with you; this is simply to help me capture all of the information that comes out of it. If you prefer, I can make written notes instead. You can ask for the audio-recorder to be switched off at any point during the interview. The recordings will be destroyed as soon as they are transcribed, the transcripts will be kept in a secure location for five years after the study is complete and then they will be destroyed.

Before we begin, I'd like to provide some ground rules for the discussion:

- You are being digitally recorded, so speak clearly;
- We will anonymise the transcript so that nobody or nowhere can be identified by name.

However, please try to avoid naming specific people or locations;

- Everything discussed here is confidential. However, if you were to reveal anything that would be considered unlawful or anything that would place you at somebody else at risk of harm, we may have to report this to a clinical supervisor or manager.

Unless you have any questions for me, then we can begin.

For peer review only - http://bmjopen.bmj.com/site/about/guidelines.xhtml

Part one: background

Can you tell me a little about your professional background and current place(s) of work?

- Name and gender
- Profession
- Years of experience
- Current job title and place of work

The CDS system Optimise Rx®

- (practice staff) how long has Optimise Rx® been installed, where is it installed
- Was anything like Optimise Rx® installed previously
- At what point did you become involved with the CDS system

For peer review only

Part two: main discussion

Questions chosen will depend upon how many interviews have been conducted with the participant and what past response(s) were, as multiple interviews may take place over time at 0-6 months, 6-18 months, and 18 months + post implementation of Optimise Rx®. Some members of staff may only be interviewed once or twice, at early or late time points.

1. *Can you describe how Optimise Rx® was introduced /continues to be used into your workplace?*
    a. Who drove things forward and how, did they involve others
    b. Roles – you and management
    c. Allocation of resources and training/support for the tool – who, what, when
    d. What went well, what could be done better

2. *What do you think Optimise Rx® is there for and how it should it be used? (changing views?)*
    a. Focus participant on prescribing and monitoring safety, and the indicators
    b. Do your colleagues share your views?
    c. Do some people know more about the tool than others? What is the impact of this
    d. Do you think that the tool has actual or potential value / benefits for your work?

3. *Please describe how Optimise Rx® is used / continues to be used by you and your colleagues as part of your work, in the context of prescribing and monitoring safety.*
    a. Where possible focus attention on prescribing safety indicators
    b. Initial views/expectations versus reality of use
    c. Have you contributed to the CDS system
    d. Frequency and nature of interaction with the tool over time
    e. Who uses the tool and what outputs are generated, are some users different

4. *Can you tell me about how Optimise Rx® 'fits in' with your existing work? (over time)*
    a. Where possible focus attention on prescribing safety indicators
    b. Has the tool changed the way you or the team works?
    c. Has the tool changed the nature of yours or others work – type, volume, order
    d. Have you or others adapted your work to suit the tool, or vice versa
    e. Has the tool become an integral part of daily work? How was this achieved
    f. Do you (still) think that using the tool should be part of your role/duties and why?
    g. Do you (still) think that this CDS system should be used routinely across primary care?

For peer review only - http://bmjopen.bmj.com/site/about/guidelines.xhtml

5. *How well does Optimise Rx® work in your practice(s)?*
    a. Is the information produced by the CDS in relation to prescribing and monitoring safety: understood, timely, relevant, useful?
    b. Have the potential benefits of the tool been realised?
    c. Are there any problems with how well the CDS system works and how are these dealt with, e.g. technical issues, interface issues
    d. Has the system disrupted working relationships?
    e. Is any information collected to find out how well the CDS system is working for you and the team? Who is responsible and how is this information used
    f. Do you have any ways in which the team get together to talk about the CDS tool and how well it is working for you? Who is responsible for driving/organising this and what have been the outputs of such activity
    g. What is your opinion of the value of the system on your work, and does this differ to the wider team?
    h. Do you have confidence in the system – what gives you confidence or takes it away
    i. Do you have confidence in others using the system, are some people more skilled than others in using it and how does this affect things
    j. Example of where the CDS has improved / struggled to improve care?
    k. Do you support the use / ongoing use of the system?

<u>Interviewer conclusion:</u>

Concluding questions

- *Is there anything that you would like to talk about?*
- *Is there anything that you would like to go back and talk about?*

Switch off tape recorder

[INTERVIEWER TO ARRANGE SUBSEQUENT INTERVIEW(S) IF REQUIRED AND PRACTICAL]

Many thanks for taking the time to help us with this study. Your contribution has been extremely valuable. If you wish we can send you a copy of your interview transcript, and you can also request to receive a summary of the findings of this research study, just ask us. In the meantime please feel free to contact either myself or the other researcher(s) involved if you have questions in future.

For peer review only - http://bmjopen.bmj.com/site/about/guidelines.xhtml

Using Sociotechnical Theory to Understand Medication Safety Work in Primary Care and Prescribers use of Clinical Decision Support: A Qualitative Study.

COREQ checklist

Note: in order to minimize the length of the manuscript, some of the details on the checklist (marked '*') are not included in the manuscript.

Guide question		*Response*	*Page number in manuscript*
1	Interviewer/facilitator	MJ conducted the interviews.	P9
2	Credentials	MJ holds an PhD in Pharmacy Practice	*
3	Occupation	MJ : Research Associate in medication safety;	*
4	Gender	MJ male	*
5	Experience and training	MJ has previous experience of undertaking qualitative research in healthcare at PhD and postdoctoral level	*
6	Relationship established	The researchers were not known to the participants prior to the study	*
7	Participant knowledge of the researcher	Participants were made aware of the reasons for doing the research via the information which was given to the participant prior to the interview	Sampling and recruitment P8; Ethics statement
8	Researcher characteristics	The researchers had identified the study topic as part of larger programmes of work in their research groups, medication safety in primary care.	*
9	Methodological orientation and theory	Sociotechnical theory	Background
10	Sampling	The sampling was purposive and included people involved in prescribing medicines who had knowledge and experience of using CDS in their daily work in general practices within the four CCG areas, including GPs, general practice nurses and general practice-based pharmacists. Sampling and recruitment of general practices was facilitated through discussions with CCG managers. MJ approached potential practices between December 2017 and November 2019, by email or telephone and invited them to take part. In addition MJ visited CCG meetings of groups of practices. After each practice had indicated that their staff might be interested in taking part, the practice was visited by MJ to further explain the study and provide written information. Practices consented to take part in the study before individual staff were approached. Individual general practice staff were approached directly by telephone or email, or through liaising with the general practice manager. A total of 41 practices were approached; nine practices consented to take part and 32 either declined to take part or did not respond.. Practices commonly gave time and staffing	P8

		commitments as reasons for not taking part. Each potential participant was provided with written information about the study and given a minimum of 24 hours to decide if they wished to take part. MJ then contacted those staff wanting to take part by telephone or email to arrange a convenient time for the interview. The interview process was two stage with follow-up interviews conducted approximately 12 months after the first interview with a convenience sample of different participants (n=7) in order to understand any changes that may have occurred and if any new themes were emerging.	
11	Method of approach	Participants were approached by telephone or email	P8-9
12	Sample size	Health professionals at 41 general practices	P8
13	Non-participation	A number of possible participants were approached but declined to participate. This was predominantly this was for reasons of time, workload or lack of use of the system.	*
14	Setting of data collection	All interviews were conducted by MJ and took place at the participants' usual place of work (general practice, CCG offices)	P9
15	Presence of non-participants	No non-participants were present	*
16	Description of sample	Twenty-three interviews were conducted with 20 participants (14 GPs, 3 nurse prescribers and 3 practice pharmacists). The first interviews were conducted between February 2018 and November 2019 with follow-up interviews conducted between August 2019 and June 2020. Interviews ranged in duration from 13-41 minutes with a mean length of 31 minutes.	P9
17	Interview guide	The semi-structured interview schedule was informed by previous studies in medication safety	P7 Appendix S1
18	Repeat interviews	7 follow-up interviews were conducted	P8
19	Audio/visual recording	Audio recording only, with consent from the participant	Ethics statement
20	Field notes	None	*
21	Duration	Interviews ranged in duration from 13-41 minutes with a mean length of 31 minutes.	P9
22	Data saturation	Data collection continued until saturation was reached and no new themes emerged from the interviews..	P9
23	Transcripts returned	No transcripts were returned to participants	*
24	Number of data coders	MJ coded the data but regular discussions codes were held with co-authors	P9-10
25	Description of the coding tree	A coding tree description is not given. Coding themes were developed from the first transcripts and then developed through discussions amongst the authors	P9-10
26	Derivation of themes	Emerging thematic codes were applied to the data and new themes emerged from the data. This is described in the analysis section	P9-10
27	Software	QSRNvivo 12 software was utilised to manage the data	P9
28	Participant checking	No	*
29	Quotations presented	Please see the results section of the manuscript	P10-P16
30	Data and findings consistent		P10-P16
31	Clarity of major themes		P10-P16
32	Clarity of minor themes		P10-P16

For peer review only

BMJ Open

Using sociotechnical theory to understand medication safety work in primary care and prescribers' use of clinical decision support: a qualitative study

Journal:	*BMJ Open*
Manuscript ID	bmjopen-2022-068798.R2
Article Type:	Original research
Date Submitted by the Author:	11-Apr-2023
Complete List of Authors:	Jeffries, Mark; The University of Manchester, NIHR Greater Manchester Primary Care Patient Safety Translational Research Centre; The University of Manchester, Centre for Pharmacoepidemiology and Drug Safety, Division of Pharmacy and Optometry, School of Health Sciences<br>Salema, Nde-Eshimuni; University of Nottingham, Centre for Academic Primary Care; The University of Manchester, NIHR Greater Manchester Primary Care Patient Safety Translational Research Centre.<br>Laing, Libby; University of Nottingham, Centre for Academic Primary Care<br>Shamsuddin, Azwa; University of Hull, School of Health Sciences<br>Sheikh, Aziz; University of Edinburgh, Division of Community Health Sciences<br>Avery, Tony; University of Nottingham, Primary Care<br>Chuter, Antony; University of Nottingham<br>Waring, Justin ; University of Birmingham<br>Keers, Richard; University of Manchester, Centre for Pharmacoepidemiology and Drug Safety ; Suicide, Risk and Safety Research Unit, Greater Manchester Mental Health NHS Foundation Trust, Manchester
**Primary Subject Heading**:	Health services research
Secondary Subject Heading:	General practice / Family practice, Qualitative research, Health informatics
Keywords:	Quality in health care < HEALTH SERVICES ADMINISTRATION & MANAGEMENT, Primary Care < Primary Health Care, QUALITATIVE RESEARCH

SCHOLARONE™
Manuscripts

I, the Submitting Author has the right to grant and does grant on behalf of all authors of the Work (as defined in the below author licence), an exclusive licence and/or a non-exclusive licence for contributions from authors who are: i) UK Crown employees; ii) where BMJ has agreed a CC-BY licence shall apply, and/or iii) in accordance with the terms applicable for US Federal Government officers or employees acting as part of their official duties; on a worldwide, perpetual, irrevocable, royalty-free basis to BMJ Publishing Group Ltd ("BMJ") its licensees and where the relevant Journal is co-owned by BMJ to the co-owners of the Journal, to publish the Work in this journal and any other BMJ products and to exploit all rights, as set out in our licence.

The Submitting Author accepts and understands that any supply made under these terms is made by BMJ to the Submitting Author unless you are acting as an employee on behalf of your employer or a postgraduate student of an affiliated institution which is paying any applicable article publishing charge ("APC") for Open Access articles. Where the Submitting Author wishes to make the Work available on an Open Access basis (and intends to pay the relevant APC), the terms of reuse of such Open Access shall be governed by a Creative Commons licence – details of these licences and which Creative Commons licence will apply to this Work are set out in our licence referred to above.

Other than as permitted in any relevant BMJ Author's Self Archiving Policies, I confirm this Work has not been accepted for publication elsewhere, is not being considered for publication elsewhere and does not duplicate material already published. I confirm all authors consent to publication of this Work and authorise the granting of this licence.

For peer review only

Using sociotechnical theory to understand medication safety work in primary care and prescribers' use of clinical decision support: a qualitative study

Mark Jeffries (corresponding author)

NIHR Greater Manchester Patient Safety Translational Research Centre, Division of Pharmacy and Optometry, School of Health Sciences, Room 1.134, Stopford Building, University of Manchester, Oxford Road, Manchester M13 9PT, UK

mark.jefferies@manchester.ac.uk

Nde-Eshimuni Salema [1, 2]

Libby Laing [1,3],

Azwa Shamsuddin [3,4]

Aziz Sheikh,[4]

Tony Avery[1, 2]

Antony Chuter [5]

Justin Waring[7]

Richard N Keers [1,7,8]

[1] NIHR Greater Manchester Primary Care Patient Safety Translational Research Centre, University of Manchester, Manchester Academic Health Sciences Centre (MAHSC), Manchester, UK

[2] Centre for Academic Primary Care, School of Medicine, University of Nottingham, Nottingham, UK

[3] School of Health Sciences, University of Hull, Cottingham Road, Hull, UK

[4]Usher Institute, University of Edinburgh, Edinburgh, UK

[5] 68 Brighton Cottages, Copyhold Lane, Lindfield, Haywards Heath, West Sussex, UK

[6]School of Social Policy, Health Services Management Centre, University of Birmingham, Birmingham, UK

For peer review only - http://bmjopen.bmj.com/site/about/guidelines.xhtml

[7] Centre for Pharmacoepidemiology and Drug Safety, Division of Pharmacy and Optometry, School of Health Sciences, University of Manchester, Manchester, UK

[8] Suicide, Risk and Safety Research Unit, Greater Manchester Mental Health NHS Foundation Trust, Manchester, UK

Word count: 5370

For peer review only

ABSTRACT

Objectives

The concept of safety work draws attention to the intentional work of ensuring safety within care systems. Clinical decision support (CDS) has been designed to enhance medication safety in primary care by providing decision-making support to prescribers. Sociotechnical theory understands that healthcare settings are complex and dynamically connected systems of fluid networks, human agents, changing relationships and social processes. This study aimed to understand the relationship between safety work and the use of CDS.

Design and setting

This qualitative study took place across nine different general practices in England. Stakeholders included general practitioners (GPs) and general practice-based pharmacists and nurse prescribers. Semi-structured interviews were conducted to illicit how the system was used by the participants in the context of medication safety work. Data analysis, conducted alongside data collection was thematic, and drew upon socio-technical theory

Participants

Twenty-three interviews were conducted with 14 GPs, three nurse prescribers and three practice pharmacists between February 2018 and June 2020.

Results

Safety work was contextually situated in a complex network of relationships. Three interconnected themes were interpreted from the data: i) the use of CDS within organisational and social practices and workflows; ii) safety work and the use of CDS within the interplay

For peer review only - http://bmjopen.bmj.com/site/about/guidelines.xhtml

between prescribers, patients and populations; and iii) the affordances embedded in CDS systems.

Conclusion

The use of sociotechnical theory here extends current thinking in patient safety particularly in the ways that safety work was co-constituted with the use of CDS alerts. This has implications for implementation and use to ensure that the contexts into which such CDS systems are implemented are taken into account. Understanding how alerts can adapt safety culture will help improve the efficacy of CDS systems, enhance prescribing safety and help to further understand how safety work is achieved in primary care.

Strengths and limitations of this study

- The use of sociotechnical theory to frame the thematic analysis enabled a nuanced understanding of the ways in which the clinical decision support was utilised for medication safety work.
- This study had a longitudinal design with a variety of stakeholders from different health professions in primary care, interviewed over time across multiple geographical locations.
- Semi-structured interviews were conducted with a full range of different prescribers in different roles (general practitioners [GPs], nurses and pharmacists).
- Many practice staff were drawn from two of the four targeted CCG areas due to recruitment difficulties related to GP time and workload.
- This study relied solely upon interview data and may have benefited from observations or other ethnographic approaches that might have uncovered 'work-as-done'.

For peer review only - http://bmjopen.bmj.com/site/about/guidelines.xhtml

INTRODUCTION

Healthcare services are increasingly interpreted as complex systems comprising fluid networks of people and technologies that interact in dynamic, non-linear and inter-dependent ways. [1-4] New thinking in the field of patient safety, often termed Safety-II, that focuses on 'work-as-done', rather than 'work-as-imagined', understands that safety is achieved less because people follow strict rules but rather because people adapt and respond to unanticipated events.[3 5] This idea departs from the 'measure and manage' orthodoxy of patient safety policies by attending to the situated practices of care work in the context of wider and inherently complex socio-cultural systems. [6 7] The concept of 'safety work' captures the idea that clinicians routinely need to engage in purposeful activities to actively create and restore safety in the context of dynamic and changeable systems.[8] Safety work has been understood from the concept of the multidimensional and dynamic safety culture of primary healthcare organisations. [9] This offers a way of exploring the interplay between system factors and also the purposeful behaviours of clinicians that goes beyond the reductionist and linear view of established safety perspectives, and instead foregrounds the combined influence of context and work as shaping clinical safety. Safety work has been seen to entail an understanding of safety culture amongst staff, [9] systems and collaboration between reception, administration and medical staff, [10] articulation work, [11] development of relationships and engagement with stakeholders [12 13] and management of workload. [8]

Sociotechnical theory, that draws upon interdependent and constructionist theories considers that people and technology are reciprocally and recursively related and as a consequence the outcomes of the relationships between the social, human agency and technology are considered as interdependent and not simply as the interactions between homogenous unique elements. [14 15] This approach focuses upon the social processes involved in the use of the

For peer review only - http://bmjopen.bmj.com/site/about/guidelines.xhtml

technology since it sees technological use and adoption as a social practice that may involve negotiation and conflict. Interdependent sociotechnical theories do not understand technology as something static that is external to social contexts but as part of that context. [16 17.] The implementation of Information Technology (IT) in health care settings has been explored from such sociotechnical perspectives.[18 19] IT does not operate in isolation from social and organisational contexts and as such plays a potential role in both adding to system complexity and hence unsafe practice, and also contributing to the safety work of people within these systems. [1 20]

Extending this view, sociomateriality is a combination of the material properties of the technology with social processes. Sociomateriality considers that properties of the technology may be fixed therefore allowing for differences in use to be assigned to such social processes and contexts. [17] Technology has been conceptualised as offering affordances in which the material properties of the technology, through interaction with human agents, allow for the possibilities for certain actions. [21-23] This contrasts to approaches that might focus narrowly and deterministically upon the functions and capacities of the technology. In the concept of affordances, technology has the possibility of both shaping human action and being shaped by different social processes and practices. [21] New rules and conventions may evolve in a dynamic interaction between the technology, users and contexts that then changes social processes and practices. [16 17] In this way technology is not a casual facilitator but affords the possibility of action as one element of a complex social process. [17 21 22]

Medications safety technologies

The World Health Organization's (WHO) Third Global Patient Safety Challenge: Medication Without Harm places medication safety as a global priority. [24] Prescribing errors in general practice are an important and expensive cause of preventable safety incidents, illness, hospitalisations and deaths. [25] Medication safety across primary care operates within complex

For peer review only - http://bmjopen.bmj.com/site/about/guidelines.xhtml

contexts that often involves collaboration between different general practice staff, between people and technology and is a fluid, negotiated, social process. [10 26 27] Prescribing errors have been considered to occur because of a multitude of error producing conditions including prescriber characteristics, patient characteristics, the working environment, the specific tasks and issues with technology. [28]

Prescribing safety in general practice is increasingly promoted through the use of specialist clinical decision support (CDS) tools used to provide clinicians with clinical knowledge and patient related information to improve the quality of prescribing and reduce medication error. [29 30] The CDS system evaluated here has been in use across general practices in England and Wales since 2014. The CDS is embedded within the GP clinical system and provides a range of patient specific messages that appear at the point of prescribing as a dialogue box or 'pop-up'. Prescribers have full discretion as to whether they accept the message. Previous evaluation of CDS systems focused upon those designed to improve care outcomes often in hospital settings. [29 31 32] CDS has been seen as moderately successful, but it is widely acknowledged that prescribers often override alerts due to high volumes and limited perceived value ('alert fatigue'), and particularly if they interrupt clinical workflow by arriving at inappropriate times in the consultation.[32-35] 'Success' or 'failure' in implementation of IT has in the past been attributed to material properties of the technology and interoperability, user characteristics, and organisational settings and processes. [36] It has however been suggested that discrete individual barriers and facilitators are less important than the complex interactions between those factors. [16 36] Engagement between the health professionals working in clinical settings and the technology may also inform understanding of the potential impact on changes in working practices, roles and responsibilities. [12 37] There has been little in the way of qualitative research that has utilised sociotechnical theory to understand how medication safety alerts might operate as part of the safety work undertaken

For peer review only - http://bmjopen.bmj.com/site/about/guidelines.xhtml

by those working within primary care. This study aimed to apply sociotechnical theory to understand the use of CDS in the accomplishment of safety work in primary care, to understand how CDS transforms safety work, and how new forms of safety work are involved in the implementation and use of CDS.

METHODS

Study design

This qualitative study used semi-structured interviews with multiple stakeholders working as prescribers in general practices in England. The study took place across nine different general practices within four separate Clinical Commissioning Groups (CCGs), in the North West and East Midlands regions of England. The study was part of a wider qualitative longitudinal process evaluation of interventions designed to improve prescribing safety in primary care.[38 39]

Sampling and recruitment of participants

The sampling was purposive and included people involved in prescribing medicines who had knowledge and experience of using CDS in their daily work in general practices within the four CCG areas, including GPs, general practice nurses and general practice-based pharmacists. Sampling and recruitment of general practices was facilitated through discussions with CCG managers. MJ approached potential practices between December 2017 and November 2019, by email or telephone and invited them to take part. In addition MJ visited CCG meetings of groups of practices. After each practice had indicated that their staff might be interested in taking part, the practice was visited by MJ to further explain the study and provide written information. Practices consented to take part in the study before individual staff were approached. Individual general practice staff were approached directly by telephone or email, or through liaising with the general practice manager. A total of 41

For peer review only - http://bmjopen.bmj.com/site/about/guidelines.xhtml

practices were approached; nine practices consented to take part and 32 either declined to take part or did not respond. Practices commonly gave time and staffing commitments as reasons for not taking part. Each potential participant was provided with written information about the study and given a minimum of 24 hours to decide if they wished to take part. MJ then contacted those staff wanting to take part by telephone or email to arrange a convenient time for the interview. The interview process was two stage with follow-up interviews conducted approximately 12 months after the first interview with a convenience sample of different participants (n=7) in order to understand any changes that may have occurred and if any new themes were emerging.

Data collection

The semi-structured interview schedule was developed by MJ and RK drawing upon previous studies in medication safety in primary care. [12 19 40] This was designed to illicit how CDS was used by the participants in the context of medication safety work (see Appendix S1). In order to ensure it remained appropriate the interview schedule was reviewed throughout data collection. Interviews continued alongside data analysis in an iterative approach until data saturation was reached. We understood data-saturation as being achieved when we judged further interpretation of codes and themes would not provide further insights. [41] All interviews were conducted by MJ and took place face-to-face at the participants' usual place of work (general practice, n=19) or by telephone (n=4); all were digitally audio-recorded. Participants were offered a £20 shopping voucher per interview as acknowledgement of their time. Twenty-three interviews were conducted with 20 participants (14 GPs, 3 nurse prescribers and 3 practice pharmacists). Follow-up interviews were conducted with 7 participants (5 GPs, 1 nurse, 1 practice pharmacist).The first interviews were conducted between February 2018 and November 2019 with follow-up interviews conducted between

For peer review only - http://bmjopen.bmj.com/site/about/guidelines.xhtml

August 2019 and June 2020. Interviews ranged in duration from 13-41 minutes with a mean length of 31 minutes.

Data analysis

Data analysis was informed by interdependent and constructionist sociotechnical theories and models. [14 15 16] MJ led the analysis, which was thematic and conducted alongside data collection following verbatim transcription. The analysis followed two parts: firstly, an inductive thematic approach informed by Braun and Clarke [42] and secondly a deductive template approach which involved developing a coding template from the first stage. [43] For the initial inductive thematic approach, MJ used QSR NVIVO 12 to organise the data and inductively coded early interviews to identify features, patterns, groups of codes and potential themes to build a preliminary framework for application to the data and to inform further data collection. These codes were discussed, in the context of sociotechnical theory, with the wider research team (RK NS LL AC ASha). These discussions led to the development of a further coding template that was applied to the full data set, refined and reworked. From this coding, the final themes as presented in the results were interpreted.

Patient and public involvement

AC is a patient and public representative who was involved in all aspects of the study and is a co-author of the paper.

RESULTS

Interpretative data analysis found a complex network of relationships between the technology, different users and the contexts in which the technology was utilised. Safety work and the use of CDS was contextually situated in this network and reciprocally interrelated with it.

Three broad, interconnected themes were interpreted from the interview data.

For peer review only - http://bmjopen.bmj.com/site/about/guidelines.xhtml

- The use of CDS within organisational and social practices and workflows;
- The use of CDS within the interplay between prescribers, patients and populations;
- Sociomateriality – the affordances embedded in CDS.

The use of CDS within organisational and social practices and workflows

For many prescribers the use of CDS reflected their established commitment to promoting safety within their practice which was described as illustrative of their a *'safety climate'*.

> *I think we are a safety conscious practice […] we don't want mistakes to happen so therefore creating a safe climate where you feel you are doing things safety is actually really important to us as a practice.* ***GP12***

Organisational culture varied. One GP reflected upon these variations across different sites of the group practice in which they worked. One site had a strong focus upon being *'traditionally good prescribers'* who had '*work(ed) very hard […] on the prescribing* ***(GP3)****.* Such 'good' prescribing was seen to be about stability which **(GP3)** suggested '*creates much improved prescribing'* in contrast to another site of their group practice which, they said, had high staff turnover. This was reflected in the number of CDS alerts seen. Having a '*safety system'* was a way of avoiding many alerts. A safety culture that included medication reviews and which had been '*done …for so long that it's relatively slick',* was perceived as conducive to utilising alerts from CDS systems in a way that avoided being overwhelmed.

> *Medication reviews I do here. This probably might explain why we've got less (alerts) here. We do a six month or annual review on people that are only on two things here, and we've done it for so long that it's relatively slick. Whereas other places where they've not had review I'm going into a jungle almost of interactions and possible problems. They've never had any safety system before, so there's lots of alerts will go on.* ***GP3***

Organisational practices involved workflows where the use of the system was most pertinent in medication reviews. The following GP utilised CDS mostly when undertaking repeat prescribing. This was perceived to lead to disregarding alerts because '*the patient's not in front of you'* and because of the volume of prescriptions that needed repeat authorisation.

> *I don't often use it (CDS support) in consultations, because I suppose that's one of the problems of it, […] the time it crops up in workflows most, is when you're signing today's 60 repeat prescriptions, so the patient's not in front of you, but there's a medication review overdue, […] (the alert) flashes up and tells you that something minor could need changing, and you sort of let it go.* ***GP13***

The use of CDS alerts within the interplay between prescribers, patients and populations

The characteristics of patients and roles of prescribers impacted upon the CDS use for medication safety. Users with differing roles in general practice might not use CDS in the same way. The wider demographics of patient groups and broader holistic needs of individual patients were considered relevant to how prescribing would be prioritised and how CDS was used. For one GP, changes to specific individual medications, as alerted by CDS, was not patient's primary concern.

> *…if you've got a patient with 20 different drugs, […] they've just lost their job, they're about to become homeless, they've got epilepsy, depression, anxiety, diabetes that's out of control, they've got 15 appointments, but they've missed the last ten, and they've got a safeguarding issue with their child […] There are much, much bigger issues for them.* ***GP5***

Individual patient needs, particularly for patients with complex medical problems or multi-morbidity, could impact upon the ways in which CDS alerts were responded to by prescribers.

For peer review only - http://bmjopen.bmj.com/site/about/guidelines.xhtml

This GP suggested that it was difficult to respond to alerts if patients were '*on long lists of medication already'* ***GP14***

Alerts were rejected if the prescriber disagreed and were considered inappropriate to the patient because of other morbidity, frailty or their social situation. Safety work was accomplished by the prescribers balancing alerts against their own knowledge and understanding of individual patient circumstances. In this specific case, leaving a patient with epilepsy without medication as the alert had suggested was perceived as less safe.

> *Sometimes it's hazardous not to prescribe [...] some of the epilepsy drugs you can't give them safely because they're dangerous drugs; and it's telling you all these warnings, but you've got to give something because it would be more dangerous to leave somebody fitting […] somebody with bad COPD (chronic obstructive pulmonary disease) and they're on a host of other drugs, there really isn't an antibiotic that you can give that doesn't have a risk of cardiac arrhythmia or something like that; (but) , I can't leave them without the antibiotic [...] we've got to give something.* ***GP1***

Safety work was also accomplished by different prescribers within the context of responsibilities, and moral and ethical behaviour. Prescribers reflected how alerts did not remove the responsibility of a prescriber's signature and prescribers still had to justify their actions.

> *I don't think anyone tool […] , you shouldn't rely on just one thing at all. You are still responsible for prescribing and what you prescribe so you've still got to justify it. You can overrule …* ***GP5***

Alerts were also seen within the context of balancing different responsibilities towards the patient, the wider practice population and the management of resources.

For peer review only - http://bmjopen.bmj.com/site/about/guidelines.xhtml

So our prime responsibility is to the patient who's in front of us now. But, at the same time, we have a responsibility to our whole practice population, and to the country, of managing resources correctly. So, we have three different responsibilities that exist, almost like a holy trinity, they're all there at the same time [...] And so anything that allows you to focus on one, while monitoring your progress on the other, is likely to be helpful, or feels helpful. So, you know, if I have someone in front of me who needs an antidepressant, and the current evidence is that sertraline is better than citalopram, but I didn't get to see that, if (CDS) comes up and says, actually we're now supposed to be giving sertraline, I think, oh that's great, 'cause that's a helpful thing, 'cause it helps me monitor my other obligations at the same time. ***GP18***

Knowing that there was patient information within alerts meant that the clinician had a moral obligation to act upon that information.

....there's a degree of responsibility, if you know that you have a tool and, you know, and there's potentially a person sat there, who could have been looked at, who hasn't been looked at, and is subject to potential harm. You've got responsibility, haven't you, as a clinician, because that information is there for you to access. ***GP15***Safety work and prescribing was undertaken differently by different prescribers. This nurse considered that whilst they had been prescribing for 11 years it was generally felt amongst nursing staff that prescribing was an "*extended role*". This led to a lack of confidence, a fear of making mistakes and the seeking of support.

I think the way nurses think because we see it as an extended role, it's not part of this. I think there's that element of it and we're always worried, we're always covering our back all the time. A doctor said to me once that it's the mind set of how we're trained, nurses

For peer review only - http://bmjopen.bmj.com/site/about/guidelines.xhtml

are trained to question, have I done something wrong […]it's how we think, scared to do something wrong. ***Nurse 1***

In contrast, the use of CDS was seen as being dependent upon experience and expertise. This GP suggested that it was easy to use and decide whether to accept alerts or not because they had many years' experience and was the prescribing lead for the practice. They suggested that they were more likely to rely upon their own experience and knowledge than less experienced prescribers who might be more likely to rely upon alerts.

Having been a GP for a long time, I'm also the prescribing lead for the practice, […] obviously, that relies on your own experience, skills, confidence, that sort of thing. I think probably as maybe one of the relatively older GPs, […] having a reasonable amount of experience and confidence in what you're doing, probably means that you cancel it off more often than a younger one. ***GP14***

For this GP there was a clear contrast between how they undertook safety work using CDS and how that was conducted by their practice pharmacist. Practice pharmacists reported that they saw patients for longer appointment times and would be more likely to be dealing with medication reviews for patients with long-term conditions. Their contrasting work practices were related to differences in response to CDS alerts with the GP less likely to be methodical and more likely to not change medications in case the patient complained.

…it's been interesting seeing how our pharmacist works, now that (they are) seeing patients and doing (their) own medication reviews. (They are) much more thorough and […] will challenge people a lot more and say, look you've been on this thing, I'm not sure you necessarily need that, or you may be need something slightly different, and will go through it in a more methodical fashion. I think we do probably in our consultations, sort of, go through things a little bit more quickly. ***GP4***

For peer review only - http://bmjopen.bmj.com/site/about/guidelines.xhtml

Sociomateriality – the affordances embedded in the CDS

CDS was used within Electronic Health Record (EHR) systems and was dynamically linked to other databases and clinical systems that could provide alerts. As a consequence, this impacted upon how CDS was used, how users interacted with it, and how the alerts were responded to. Part of this was associated with a frustration about what different technologies could or could not do, or what could or could not be done with different technologies. Prescribers talked of CDS when embedded with one particular clinical system *'failing to launch' …'it's just so many more clicks on particular things of your screen'* ***GP12*** or '*the number of warnings that comes up in (name of clinical system) is far too many'* ***GP13.*** This was said to mean *'it slows down and frustrates you as you're doing an important task but at the same time you're not getting the advice from it as well.'* ***GP13***. Affordance in this way was not just about non-use or abandonment but about how that made users perceive the system and feel about using it, which then in turn led to a negative response. Such frustration with the CDS could lead to important medication safety issues.

> *We had a significant event where a medication was prescribed to a patient that shouldn't have been. We know that at least one of the factors in that was the fact that in the set up process of (the clinical system) different doctors chose different security settings for medicines safety…* ***GP13***

The perception that CDS was not providing accurate and useful alerts could mean that the system was ignored and therefore safety work potentially compromised.

In contrast, some participants described how CDS provided affordances for prescribers to undertake safety work. This GP considered the alerts not intrusive, easily overridden and providing useful and sensible safety advice. Such safety advice was seen as important since it could mitigate against safety problems for patients in the future.

For peer review only - http://bmjopen.bmj.com/site/about/guidelines.xhtml

I don't find them too intrusive really. I mean it is another thing that happens that didn't happen before but it's as we discussed before, it was they are relatively easy to override and the suggestions that are being made generally feel quite sensible [...] And if it's a safety thing, then generally I've been quite pleased that it's happened because it's given me an opportunity to make a change before potentially something that might cause me a problem later down the line. ***GP4 (Follow-up interview)***

CDS was seen as providing prompts to safer prescribing that challenged prescribers to think about what they were about to prescribe. This could take the form, as described by this GP of avoiding a prescribing error.

… today it's prompted me not to prescribe the wrong dose of oxycodone. So off a picking list I was close to picking the wrong strengths of oxycodone. And that came up with an (CDS) prompt which was great. […] , so I was very happy to have something held up in front of my face going, are you sure about that? ***GP4***

DISCUSSION

The use of CDS for medication safety in primary care was seen to be contextually situated in the social practices of safety work. This builds upon understandings of healthcare settings as complex systems with different characteristics including people, tasks and technology. [3] In this study, safety work was accomplished within safety cultures, which varied for patients and prescribers and therefore shaped their particular forms of engagement with the CDS and safety work. The use of sociotechnical theory here extends current thinking in patient safety particularly in the ways that safety work was co-constituted with the use of CDS alerts.

Safety work in primary care

The safety work undertaken by prescribers when using CDS was often shaped by the perceived health needs and broader characteristics of the patient. Where CDS support was less easy to

For peer review only - http://bmjopen.bmj.com/site/about/guidelines.xhtml

accommodate to the holistic practices of prescribers, in terms of their relationship with patients, it was typically underutilised. Specifically, for patients with multiple and complex care needs, prescribers suggested it was necessary to consider factors other than prescribing, and relatedly the existing CDS system presented functional barriers to working with patients on multiple medicines. A recent systematic review and meta-analysis of medication harm across health settings found the highest incidence of medication harm in patients with high comorbidity and related polypharmacy. [44] It has previously been suggested that decision-making is made more complicated for clinicians when dealing with patients with multi-morbidity. [45 46] As such, the under-utilisation of CDS might be seen as stemming from a lack of appreciation amongst designers and policymakers about how prescribing safety is shaped by the particular relationship between prescriber and patient, in the context of the former's prevailing safety practices and the latter's particular health needs.

We found that the utilisation of CDS for safety work within primary care was linked to the affordances within the technology and significantly how these related to the perceived needs of the patient and the associated approach to medical decision-making. That is, GPs' safety work often involved ignoring alerts where they were perceived as inaccurate, overwhelming, or irrelevant to the complexity of the decision-making for a given patient. Although our study did find that in other relational contexts, i.e. with less complex patients, or where there was more time available such as in consultations with practice pharmacists, CDS did provide prescribers with valued and useful alerts that were seen as enhancing their safety work.

CDS alerts were not neutral in that they provided a specific recommendation to the prescriber. This could be seen as form of 'digital nudging' whereby technology might be seen to influence choices and 'nudge' people into specific behaviours. [47] That the prescribers here did reject alerts suggests that this was an active rather than passive response to the alert CDS alerts afforded the possibility of making prescribing changes but were only acted upon if the

For peer review only - http://bmjopen.bmj.com/site/about/guidelines.xhtml

prescriber considered it appropriate within the given contexts of their practice. The system provided prompts and challenges in the form of alerts, which could be accepted, and acted upon in different ways, or ignored and thus there was a recursive relationship between people and the technology. [14] Responding to CDS alerts was therefore, not a linear and binary process but one that was fluid and contextually bound. Prescribers' responses to safety alerts were based upon their expertise and knowledge. Previously Swinglehurst [10] found that safety work in repeat prescribing was embedded in different types of organisational structures. This study reinforces the sense that primary care and medication safety operate as complex systems with multiple and interconnected parts. [1] We found that the organisational culture and what prescribers referred to as a 'safety climate' or 'safety culture' of particular importance with organisational practices such as workflows and tasks shaping the use of CDS. This reinforces that safety work is a social process operating within multidimensional and dynamic systems.[8 9 48] As well as organisational processes shaping the use of CDS, CDS alerts shaped the way prescribers accomplished safety work as they variously adapted their clinical practices and responded differently to them. [1 3] This included prescribers reflecting upon their moral and ethical responsibilities towards their patients and the wider community. This further highlights how safety work requires a flexible approach that is adaptive and responsive to different contextual situations and does not over rely upon technology. [10] Our study builds upon understandings of alert fatigue in that underutilisation cannot be therefore simply seen in terms of the volume and relevant of alerts but that alerts are part of the social process of managing safety within complex work in primary care.

Strengths and limitations

A particular strength of this study is the focus upon sociotechnical theory, which enabled a nuanced understanding of the ways in which the CDS was utilised. By using this theoretical lens we could further understand how the CDS alerts and those already embedded in the EHR

For peer review only - http://bmjopen.bmj.com/site/about/guidelines.xhtml

were utilised within the context of organisational culture, and prescribers workflows. This builds upon the sociotechnical theory particularly that around the concept of affordances within technology that enable human actions.[21 22] Additionally, a further strength was the longitudinal design with a variety of stakeholders from different professions interviewed over time across multiple geographical locations. A full range of different prescribers in different roles (GPs, nurses and pharmacists) were interviewed but there could have been greater breadth across general practices. The present study found some variation between different prescribers (Pharmacists, GPs or Nurses) in the ways they adopted the CDS for safety work, but we could only explore this in a limited way given our study sample. Further understanding how safety work is accomplished in the context of different users of the technology in future research would be useful. Many practice staff were also drawn from two of the four targeted CCG areas due to recruitment difficulties related to GP time and workload. Furthermore, interviewing patients to gain their perspective may well have been useful. This study also relied solely upon interview data and may have benefited from observations or other ethnographic approaches that might have uncovered 'work-as-done'. [5]

Implications for future research, policy and practice

CDS systems for medication safety have been utilised in primary care with varying success. [31 34 49] Since CDS systems operate within other technology such as the EHR it may be important to understand how different technologies interact and offer different ways people can engage in safety activities. Understanding open systems and the complexity in healthcare has been highlighted as important in health care evaluation. [1 50] Investment into CDS systems needs to be judged against how those systems will be used not just from a functionality aspect but also from the view of who the users will be and for which individual and groups of patients they will utilising the system for. How CDS are utilised for patients with multi-morbidity and polypharmacy will be an important consideration here. An important finding of our study was

For peer review only - http://bmjopen.bmj.com/site/about/guidelines.xhtml

that functionality was linked to patient characteristics and prescriber motivations in that where multiple alerts were received owing to patients having polypharmacy the system was then underutilised and the alerts could be ignored. Since this group of patients is at most risk from a prescribing error, this has important implications for practice. Multidisciplinary reviews of patients with polypharmacy have been found useful in other contexts including care homes. [51 52]

There is a recursive interrelationship between CDS alerts and safety culture in that CDS can enhance safety culture but may also be utilised differently within existing safety cultures as prescribers adapt their practices. [1 3] Further research could valuably explore how technology to enhance medication safety can shape organisational safety work and not just be utilised within a safety climate but be instrumental in building that safety culture. Understanding how alerts can adapt that safety culture and how prescribers may adapt to the alerts to form new emerging safety cultures is a challenge that will help improve the efficacy of CDS systems, enhance prescribing safety and help to further understand how safety work is achieved in primary care.

CONCLUSIONS

Sociotechnical theory enabled us to understand how the use of CDS for medication safety in primary care is context bound and dependent upon a complex and reciprocal network of relationships between the technology, different users and the places and spaces in which the technology was utilised. Where prescribers perceived their practices to have a safety culture this could impact upon the utilisation of CDS. The use of CDS for medication safety work was dependent upon who responded to alerts and for which patients the alerts were received. Since alerts for patients with multimorbidity and polypharmacy were reported to be underutilised an important implication and strategy of our findings is how prescribers may adapt their practices to ensure medication safety for this group of patients Prescribers adaptation of their practices in response to alerts has implications for the use of decision

For peer review only - http://bmjopen.bmj.com/site/about/guidelines.xhtml

support to ensure that the contexts into which it is implemented and therefore the ways in which safety work is accomplished are taken into account.

Contributors

TA secured funding. MJ, RK, TA and JW advanced the idea for the study. RK and TA provided study management. MJ collected the data. MJ led on data analysis with contributions from NS, LL, AC, RK, ASHE and ASHA. MJ drafted the manuscript which was reviewed and commented upon by all authors. All authors approved the final manuscript.

Funding

This study was part of the NIHR PRoTeCT Programme grant. PRoTeCT is funded by the NIHR Programme Grants for Applied Research Programme (1214-20012). The views expressed are those of the author(s) and not necessarily those of the NIHR or the Department of Health and Social Care. Additionally, MJ, RK, TA, LL and NS received funding from the National Institute for Health Research through the Greater Manchester Patient Safety Translational Research Centre (NIHR Greater Manchester PSTRC) grant number PSTRC-2016-003.

Competing interests

The authors declare they have no competing interests.

Ethical approval and consent to participate

Ethical approval for the study was granted by the University of Manchester Research Ethics Committee. (Ref: 2017-0842-3977). Further approval to conduct the research was granted by the NHS Health Research Authority (IRAS project ID: 233079) and by the research and development offices for the NHS participating regions. General practices gave written informed consent for their staff to be approached to take part in the study. All interview participants gave written informed consent to take part in the study, and for interviews to be

For peer review only

For peer review only - http://bmjopen.bmj.com/site/about/guidelines.xhtml

audio-recorded, transcribed verbatim and for anonymised quotations to be used in publications.

Data availability statement

This is a qualitative study confined to relatively small groups of health care professionals in specific roles, particularly GP staff. Making the full transcripts publicly available could therefore potentially lead to the identification of participants. Our ethics approval was granted based on the anonymity of the individuals consenting to participate and specifically referred to only anonymised quotations being used in reports. As such, the participants did not consent to full their transcript being made publicly available. Therefore, data cannot be made publicly available.

REFERENCES

1. Greenhalgh T Papoutsi C. Studying complexity in health services research: desperately seeking an overdue paradigm shift. *BMC Medicine* 2018;16:95.

2. Lipsitz LA. Understanding health care as a complex system. *JAMA* 2012;308(3):243-244.

3. McNab D, McKay J, Shorrock S, *et al.* Development and application of 'systems thinking' principles for quality improvement. *BMJ Open Quality* 2020;9e000714.

4. Pype P, Mertens F, Helewaut F, *et al.* Health care teams as complex adaptive systems: understanding team behaviour through team members perception of interpersonal interaction. *BMC Health Services Research* 2018;18:570.

5. Hollnagel E, Wears RL, Braithwaite J. From Safety-I to Safety-II: A White Paper. The Resilient Health Care Net. Published simultaneously by the University of Southern Denmark, University of Florida, USA, and Macquarie University, Australia, 2015.

For peer review only - http://bmjopen.bmj.com/site/about/guidelines.xhtml

6. Rowley E, Waring J. *A socio-cultural perspective on patient safety.* Boca Raton, FL, London, NewYork: CRC Press, 2017.

7. Wears RL, Sutcliffe KM. *Still not Safe: Patient Safety and The Middle-Managing of American Medicine.* New York: OUP, 2020.

8. Daker-White G, Hays R, Blakeman T *et al*. Safety work and risk management as burdens of treatment in primary care : insights from a focused ethnographic study of patients with multimorbidity. *BMC Family Practice* 2018;19(155).

9. Kirk S, Parker D, Claridge T, *et al.* Patient safety culture in primary care: developing a theoretical framework for practical use *Quality and Safety in Health Care* 2007;16:313-320.

10. Swinglehurst D, Greenhalgh T, Russell J, *et al.* Receptionist input to quality and safety in repeat prescribing in UK general practice: ethnographic case study. *BMJ.* 2011;343:d6788.

11. Grant S, Mesman J, Guthrie B. Spatio-temporal elements of articulation work in the achievement of repeat prescribing safety in UK general practice. *Sociology of Health & Illness* 2016;38(2):306-324

12. Jeffries M, Keers RN, Phipps DL *et al.* Developing a learning health system: insights from a qualitative process evaluation of a pharmacist-led electronic audit and feedback intervention to improve medication safety in primary care. *PLoS ONE* 2018;13(10):e0205419

13. Jeffries M , Keers RN, Belither H, Sanders C, Gallacher K, Alqenae F, Ashcroft DM. Understanding the implementation, impact and sustainable use of an electronic pharmacy referral service at hospital discharge: A qualitative evaluation from a sociotechnical perspective. PLoS One. 2021 Dec 22;16(12):e0261153.

14. Orlikowski WJ, Scott SV. Sociomateriality: challenging the separation of technology, work and organization. *Academy of Management Annals* 2008;2(1):433-474.

For peer review only - http://bmjopen.bmj.com/site/about/guidelines.xhtml

15 Greenhalgh T, Shaw S, Wherton J *et al*. SCALS: a fourth generation study of assisted living technologies in their organisational, social, political and policy context. *BMJ Open.* 2016;6:e010208.

16. Klecun E. Transforming healthcare: Policy discourses of IT and patient-centred care. *European Journal of Information Systems* 2016;25:64-76.

17. Leonardi PM. 'Materiality, Sociomateriality and Sociotechnical systems: What do these terms mean? How are they different? Do we need them?' in Leonardi PM, Nardi BA, Kallinikos J. eds. *Materiality and Organizing: Social Interaction in a Technological World.* Oxford: OUP, 2012 pp.25-48.

18 Greenhalgh T, Stones R, Swinglehurst D. Choose and Book: a sociological analysis of 'resistance' to an expert system. *Social Science & Medicine* 2014;104:210-19.

19. Jeffries M, Phipps D, Howard RL, *et al.* Understanding the implementation and adoption of an information technology intervention to support medicine optimisation in primary care: qualitative study using strong structuration theory. *BMJ Open* 2017;7:e014810

20. Timmermans S, Berg M. The Practice of Medical Technology. *Sociology of Health and Illness,* 2003;25, 97-114.

21. Hutchby I. Technologies, texts and affordances. *Sociology* 2001;*35*(2):441-456.

22. Petrakaki D, Klecun E, Cornford T. Changes in healthcare professional work afforded by technology: The introduction of anational electronic patient record in an English hospital. *Organization* 2016;23(2):206-226.

23. Petrakaki, D, Waring J. Barber N. Technological affordances of risk and blame: the case of the electronic prescription service in England. *Sociology of Health and Illness* 2014;36(5):703-718.

For peer review only - http://bmjopen.bmj.com/site/about/guidelines.xhtml

24World Health Organization Medication Without Harm - Global Patient Safety Challenge on Medication Safety. Geneva: WHO, 2017

25 Elliott RA, Camacho E, Jankovic D, *et al.* Economic analysis of the prevalence and clinical and economic burden of medication error in England. *BMJ Quality & Safety* 2021;30:96–105.

26. Allen D, Braithwaite J, Sandall J *et al.* Towards a sociology of healthcare safety and quality. *Sociology of Health and Illness* 2016; 38(2):181-197.

27. Jeffries M, Gude WT, Keers RN, *et al.* Understanding the utilisation of a novel interactive electronic medication safety dashboard in general practice: a mixed methods study. *BMC Medical Informatics and Decision Making,* 2020;20(69)

28. Slight SP, Howard R, Ghaleb M, *et al.* The cause of prescribing errors in English general practices: a qualitative study. *British Journal of General Practice* 2013;63:e713-e720

29. Scott IA, Pillans PI, Barra M, *et al.* Using EMR-enabled computerized decision support systems to reduce prescribing of potentially inappropriate medications : a narrative review. *Therapeutic Advances in Drug Safety* 2018;9(9):559-573.

30. Silsand L, Ellingsen G. Complex decision making in clinical practice .Computer-Supported Cooperative Work February 27th-March 2nd 2016, San Francisco CA, USA.

31. Jia P, Zhang L, Chen J, *et al.* The effects of Clinical Decision Support Systems on Medication Safety: An Overview. *PLoS ONE* 2016;11(12):e0167683.

32. Nanji KC, Slight SP, Seger DL, *et al.* Overrides of medication-related clinical decision support alerts in outpatients. *JAMA* 2014;21:487-491.

For peer review only - http://bmjopen.bmj.com/site/about/guidelines.xhtml

33. Avery AJ, Savelyich BSP, Sheikh A, *et al.* Identifying and establishing consensus on the most important safety features of GP computer systems: e-Delphi study. *Informatics in Primary Care,* 2005;13(1)3-12.

34. Hayward J, Thomson F, Milne H, *et al* "Too much, too late": mixed methods multi-channel video recording study of computerized decision support systems and GP prescribing *Journal of the American Medical Informatics Association* 2013;20(e1):e76-e84.

35. Van der Sijs H, Aarts J, Van Gelder T, *et al.* Overriding of Drug Safety Alerts in Computerized Physician Order Entry. *JAMA* 2006;13:138–147.

36. Greenhalgh T, Wherton J, Papoutsi C, *et al*. Beyond adoption: A new framework for theorizing and evaluation nonadoption, abandonment and challenges to the scale -up, spread, and sustainability of health care technologies. *Journal of Medical Internet Research,* 2017;19(11):e367 p1.

37.Mair F, May C, O'Donnell C, *et al.* Factors that promote or inhibit the implementation of e-health systems: an explanatory systematic review. *Bulletin of the World Health Organisation* 2012;90(3):57–364.

38 Jeffries M, Salema N, Laing L. *et al.* The Implementation, Use and Sustainability of a Clinical Decision Support System for Medication Optimisation in Primary Care: A Qualitative Evaluation *PLoS ONE* 2021;16(5): e0250946.

39. Shamsuddin A, Jeffries M, Keers RN, *et al.* Strategies for success of interventions to support prescribing safety in primary care. *BJGP Open.* 2021;5(5):

40. Cresswell KM, Sadler S, Rodgers S *et al.* An embedded longitudinal multi-faceted qualitative evaluation of a complex cluster randomized controlled trial aiming to reduce clinically important errors in medicines management in general practice. *Trials* 2012;13:78.

For peer review only

For peer review only - http://bmjopen.bmj.com/site/about/guidelines.xhtml

41. Braun V, Clark V. To saturate or not to saturate? Questioning data saturation as a useful concept for thematic analysis and sample-size rationales. *Qualitative Research in Sport, Exercise and Health* 2021;13(2):201-216.

42. Braun V, Clark V. Using thematic analysis in psychology. *Qualitative Research in Psychology* 2006;3(2):77-101.

43 King N. 'Doing template analysis', in Symon G, Cassell C (eds.) *Qualitative Organizational Research: Core Methods and Current Challenges.* London: Sage, 2012 pp256-70.

44. Hodkinson A, Tyler N, Ashcroft, DM, *et al.* Preventable medication harm across health care settings: a systematic review and meta-analysis. *BMC Medicine* 2020;18:313.

45. Bower P, Macdonald W, Harkeness E, *et al*. Multimorbidity, service organisation and clinical decision making in primary care : a qualitative study. *Family Practice* 2011;*28(5):* 579-87.

46. Moffat K, Mercer SW. Challenges of managing people with multimorbidity in today's healthcare systems. *BMC Family Practice* 2015;16(10):129.

47 Weinmann, M, Schneider C, vom Brocke J. Digital nudging Business Information and Systems Engineering 2016;58(6):433-436

48Waring J, Allen D, Braithwaite J, *et al*. Healthcare quality and safety: a review of policy, practice and research. *Sociology of Health and Illness* 2015;38(2):198-215

49. Trinkley KE, Blakeslee WW, Matlock DD, *et al.* Clinician preferences for computerised clinical decision support medications in primary care: a focus group study. *BMJ Health Care Informa*tics 2019;26.

For peer review only - http://bmjopen.bmj.com/site/about/guidelines.xhtml

50.Long KM, McDermott F, Meadows GN. Being pragmatic about healthcare complexity: our experiences applying complexity theory and pragmatism to health services research. *BMC Medicine* 2018;*16*(94).

51. Baqir W, Barrett S, Desai N, *et al*. A clinic-ethical framework for multidisciplinary review of medication in nursing homes. *BMJ Quality Improvement Reports* 2016;u203261 w2538.

52. Baqir W, Hughes J, Jones T, *et al*. Impact of medication review, within a shared decision-making framework, on deprescribing in people living in care homes. *European Journal of Hospital Pharmacy* 2017;24:30-33.

For peer review only

INTERVIEW SCHEDULE

Interviewer introduction:

I am [….] and I am a researcher at The University of Manchester. We are carrying out a project looking at how the clinical decision support (CDS) software tool Optimise Rx® is introduced and used over time in general practices. We are particularly interested in the role of this tool in the safe prescribing and monitoring of medications as it incorporates 'prescribing safety indicators' [describe them]. We would like to find out more about the experiences of primary care staff, managers and software developers who have worked with the Optimise Rx® system in some way.

This interview will last for up to one hour, but if this is not your first interview with us it may take more like 30 minutes. During our time together I'd like to discuss your experiences of Optimise Rx® in practice, with a focus on medication safety practices. If this is not your first interview, I would like to revisit some of the things you said last time for an update, whilst also exploring any new topics that you want to raise today. I should remind you that the interviews are confidential. I'd like to audio record the discussion if that is okay with you; this is simply to help me capture all of the information that comes out of it. If you prefer, I can make written notes instead. You can ask for the audio-recorder to be switched off at any point during the interview. The recordings will be destroyed as soon as they are transcribed, the transcripts will be kept in a secure location for five years after the study is complete and then they will be destroyed.

Before we begin, I'd like to provide some ground rules for the discussion:

- You are being digitally recorded, so speak clearly;
- We will anonymise the transcript so that nobody or nowhere can be identified by name. However, please try to avoid naming specific people or locations;
- Everything discussed here is confidential. However, if you were to reveal anything that would be considered unlawful or anything that would place you at somebody else at risk of harm, we may have to report this to a clinical supervisor or manager.

Unless you have any questions for me, then we can begin.

For peer review only - http://bmjopen.bmj.com/site/about/guidelines.xhtml

Part one: background

Can you tell me a little about your professional background and current place(s) of work?

- Name and gender
- Profession
- Years of experience
- Current job title and place of work

The CDS system Optimise Rx®

- (practice staff) how long has Optimise Rx® been installed, where is it installed
- Was anything like Optimise Rx® installed previously
- At what point did you become involved with the CDS system

For peer review only

For peer review only - http://bmjopen.bmj.com/site/about/guidelines.xhtml

Part two: main discussion

Questions chosen will depend upon how many interviews have been conducted with the participant and what past response(s) were, as multiple interviews may take place over time at 0-6 months, 6-18 months, and 18 months + post implementation of Optimise Rx®. Some members of staff may only be interviewed once or twice, at early or late time points.

1. *Can you describe how Optimise Rx® was introduced /continues to be used into your workplace?*
    a. Who drove things forward and how, did they involve others
    b. Roles – you and management
    c. Allocation of resources and training/support for the tool – who, what, when
    d. What went well, what could be done better

2. *What do you think Optimise Rx® is there for and how it should it be used? (changing views?)*
    a. Focus participant on prescribing and monitoring safety, and the indicators
    b. Do your colleagues share your views?
    c. Do some people know more about the tool than others? What is the impact of this
    d. Do you think that the tool has actual or potential value / benefits for your work?

3. *Please describe how Optimise Rx® is used / continues to be used by you and your colleagues as part of your work, in the context of prescribing and monitoring safety.*
    a. Where possible focus attention on prescribing safety indicators
    b. Initial views/expectations versus reality of use
    c. Have you contributed to the CDS system
    d. Frequency and nature of interaction with the tool over time
    e. Who uses the tool and what outputs are generated, are some users different

4. *Can you tell me about how Optimise Rx® 'fits in' with your existing work? (over time)*
    a. Where possible focus attention on prescribing safety indicators
    b. Has the tool changed the way you or the team works?
    c. Has the tool changed the nature of yours or others work – type, volume, order
    d. Have you or others adapted your work to suit the tool, or vice versa
    e. Has the tool become an integral part of daily work? How was this achieved
    f. Do you (still) think that using the tool should be part of your role/duties and why?
    g. Do you (still) think that this CDS system should be used routinely across primary care?

For peer review only - http://bmjopen.bmj.com/site/about/guidelines.xhtml

5. *How well does Optimise Rx® work in your practice(s)?*
    a. Is the information produced by the CDS in relation to prescribing and monitoring safety: understood, timely, relevant, useful?
    b. Have the potential benefits of the tool been realised?
    c. Are there any problems with how well the CDS system works and how are these dealt with, e.g. technical issues, interface issues
    d. Has the system disrupted working relationships?
    e. Is any information collected to find out how well the CDS system is working for you and the team? Who is responsible and how is this information used
    f. Do you have any ways in which the team get together to talk about the CDS tool and how well it is working for you? Who is responsible for driving/organising this and what have been the outputs of such activity
    g. What is your opinion of the value of the system on your work, and does this differ to the wider team?
    h. Do you have confidence in the system – what gives you confidence or takes it away
    i. Do you have confidence in others using the system, are some people more skilled than others in using it and how does this affect things
    j. Example of where the CDS has improved / struggled to improve care?
    k. Do you support the use / ongoing use of the system?

Interviewer conclusion:

Concluding questions

- *Is there anything that you would like to talk about?*
- *Is there anything that you would like to go back and talk about?*

Switch off tape recorder

[INTERVIEWER TO ARRANGE SUBSEQUENT INTERVIEW(S) IF REQUIRED AND PRACTICAL]

Many thanks for taking the time to help us with this study. Your contribution has been extremely valuable. If you wish we can send you a copy of your interview transcript, and you can also request to receive a summary of the findings of this research study, just ask us. In the meantime please feel free to contact either myself or the other researcher(s) involved if you have questions in future.

For peer review only - http://bmjopen.bmj.com/site/about/guidelines.xhtml

For peer review only

Using Sociotechnical Theory to Understand Medication Safety Work in Primary Care and Prescribers use of Clinical Decision Support: A Qualitative Study.

COREQ checklist

Note: in order to minimize the length of the manuscript, some of the details on the checklist (marked '*') are not included in the manuscript.

Guide question		*Response*	*Page number in manuscript*
1	Interviewer/facilitator	MJ conducted the interviews.	P9
2	Credentials	MJ holds an PhD in Pharmacy Practice	*
3	Occupation	MJ : Research Associate in medication safety;	*
4	Gender	MJ male	*
5	Experience and training	MJ has previous experience of undertaking qualitative research in healthcare at PhD and postdoctoral level	*
6	Relationship established	The researchers were not known to the participants prior to the study	*
7	Participant knowledge of the researcher	Participants were made aware of the reasons for doing the research via the information which was given to the participant prior to the interview	Sampling and recruitment P8; Ethics statement
8	Researcher characteristics	The researchers had identified the study topic as part of larger programmes of work in their research groups, medication safety in primary care.	*
9	Methodological orientation and theory	Sociotechnical theory	Background
10	Sampling	The sampling was purposive and included people involved in prescribing medicines who had knowledge and experience of using CDS in their daily work in general practices within the four CCG areas, including GPs, general practice nurses and general practice-based pharmacists. Sampling and recruitment of general practices was facilitated through discussions with CCG managers. MJ approached potential practices between December 2017 and November 2019, by email or telephone and invited them to take part. In addition MJ visited CCG meetings of groups of practices. After each practice had indicated that their staff might be interested in taking part, the practice was visited by MJ to further explain the study and provide written information. Practices consented to take part in the study before individual staff were approached. Individual general practice staff were approached directly by telephone or email, or through liaising with the general practice manager. A total of 41 practices were approached; nine practices consented to take part and 32 either declined to take part or did not respond.. Practices commonly gave time and staffing	P8

For peer review only

		commitments as reasons for not taking part. Each potential participant was provided with written information about the study and given a minimum of 24 hours to decide if they wished to take part. MJ then contacted those staff wanting to take part by telephone or email to arrange a convenient time for the interview. The interview process was two stage with follow-up interviews conducted approximately 12 months after the first interview with a convenience sample of different participants (n=7) in order to understand any changes that may have occurred and if any new themes were emerging.	
11	Method of approach	Participants were approached by telephone or email	P8-9
12	Sample size	Health professionals at 41 general practices	P8
13	Non-participation	A number of possible participants were approached but declined to participate. This was predominantly this was for reasons of time, workload or lack of use of the system.	*
14	Setting of data collection	All interviews were conducted by MJ and took place at the participants' usual place of work (general practice, CCG offices)	P9
15	Presence of non-participants	No non-participants were present	*
16	Description of sample	Twenty-three interviews were conducted with 20 participants (14 GPs, 3 nurse prescribers and 3 practice pharmacists). The first interviews were conducted between February 2018 and November 2019 with follow-up interviews conducted between August 2019 and June 2020. Interviews ranged in duration from 13-41 minutes with a mean length of 31 minutes.	P9
17	Interview guide	The semi-structured interview schedule was informed by previous studies in medication safety	P7 Appendix S1
18	Repeat interviews	7 follow-up interviews were conducted	P8
19	Audio/visual recording	Audio recording only, with consent from the participant	Ethics statement
20	Field notes	None	*
21	Duration	Interviews ranged in duration from 13-41 minutes with a mean length of 31 minutes.	P9
22	Data saturation	Data collection continued until saturation was reached and no new themes emerged from the interviews..	P9
23	Transcripts returned	No transcripts were returned to participants	*
24	Number of data coders	MJ coded the data but regular discussions codes were held with co-authors	P9-10
25	Description of the coding tree	A coding tree description is not given. Coding themes were developed from the first transcripts and then developed through discussions amongst the authors	P9-10
26	Derivation of themes	Emerging thematic codes were applied to the data and new themes emerged from the data. This is described in the analysis section	P9-10
27	Software	QSRNvivo 12 software was utilised to manage the data	P9
28	Participant checking	No	*
29	Quotations presented	Please see the results section of the manuscript	P10-P16
30	Data and findings consistent		P10-P16
31	Clarity of major themes		P10-P16
32	Clarity of minor themes		P10-P16

For peer review only
